# Safety profile of camrelizumab: An analysis based on literature and database review

**DOI:** 10.1371/journal.pone.0354252

**Published:** 2026-07-23

**Authors:** Yi Huang, Wei Li

**Affiliations:** Department of Pharmacy, Xiangtan Central Hospital (The Affiliated Hospital of Hunan University), Xiangtan, China; Deccan School of Pharmacy, INDIA

## Abstract

**Objective:**

Real-world studies on the safety of camrelizumab are scarce. This study aimed to investigate the adverse drug reactions (ADRs) associated with camrelizumab and evaluate their clinical characteristics and management.

**Methods:**

We retrieved data on ADRs related to the PD-1 inhibitor camrelizumab from the World Health Organization (WHO) adverse event reporting system database (VigiAccess) for the period from June,2019 to July 2025. Additionally, we conducted a retrospective analysis of case reports and case series on camrelizumab-related ADRs published from 2019 to 2025.

**Results:**

601 ADR reports were included in the VigiAccess database. Asian patients accounted for 99% (597/601), and the majority were male (69%). The most common classification of systemic organs (SOC) is hematological disorders (21.8%), skin reactions (14.3%), and systemic symptoms (8.8%). The main adverse reactions were: Hematological system: bone marrow suppression (10.3%), thrombocytopenia (5.1%); Skin: rash (5.7%), pruritus (4.3%), reactive capillary hyperplasia (RCCEP); systemic: fever (2.6%), chest pain (2.1%); serious events: myocarditis (1.2%), toxic epidermal necrolysis (TEN). Literature analysis included 80 patients (from China), with a median age of 60 years (range 20–84), and 72.5% were male. The main indications are non-small cell lung cancer (20%), nasopharyngeal carcinoma (20%) and hepatocellular carcinoma (11.3%). The median occurrence time of adverse reactions was 8 weeks (ranging from 10 minutes to 88 weeks). Typical ADRs include: cutaneous toxicity (45 cases, 56.3%); RCCEP (32 cases), Stevens-Johnson (SJS)/TEN (5 cases); hematological toxicity (38 cases, 47.5%): bone marrow suppression (22 cases), thrombocytopenia (16 cases); cardiotoxicity (13 cases, 16.25%), mainly myocarditis; Others: immune hepatitis (6 cases), thyroid dysfunction (5 cases).

**Conclusion:**

The ADRs of camrelizumab are primarily hematologic and skin toxicities, with a need to be vigilant for late-onset serious events (e.g., myocarditis, TEN). Early identification and corticosteroid intervention are key management strategies.

## 1 Introduction

The primary treatment modalities for malignant tumors encompass surgical resection, radiotherapy, and pharmacological interventions. Pharmacotherapy includes conventional chemotherapeutics and novel antitumor agents. Among these, immune checkpoint inhibitors (ICIs), particularly those targeting the programmed cell death protein 1 (PD-1)/programmed cell death ligand 1 (PD-L1) pathway, represent a groundbreaking advancement in tumor immunotherapy with promising therapeutic prospects. By restoring anti-tumor immunity, ICIs have demonstrated significant clinical benefits and improved overall survival (OS) in patients with various malignancies.[1]

As PD-1/PD-L1 inhibitors gain widespread clinical adoption, reports of associated adverse drug reactions (ADRs) have increased substantially. Unlike cytotoxic chemotherapy, ICI-induced ADRs predominantly manifest as immune-related adverse events (irAEs), affecting diverse organ systems including the skin, gastrointestinal tract, and endocrine glands.[2] Some systematic reviews on ICIs as a class have summarized general irAE profiles, real-world evidence focusing on the safety of individual PD-1 inhibitors, especially those with unique ADR spectra, remains limited.

Camrelizumab, a humanized anti-PD-1 monoclonal antibody, has been approved for treating multiple malignancies such as classical Hodgkin’s lymphoma, hepatocellular carcinoma, non-squamous non-small cell lung cancer, esophageal squamous cell carcinoma, and nasopharyngeal carcinoma. Beyond common irAEs (e.g., hematological and skin toxicities), camrelizumab is distinguished by a unique adverse reaction profile, notably the frequent occurrence of reactive cutaneous capillary endothelial proliferation (RCCEP), a distinctive cutaneous manifestation rarely reported with other PD-1 inhibitors. Additionally, delayed severe events such as myocarditis (reported incidence ~1.2%) and toxic epidermal necrolysis (TEN) warrant heightened clinical vigilance.[3]These idiosyncratic risks underscore the need for a dedicated safety assessment of camrelizumab, which remains underexplored compared to broader ICI reviews.

Against this backdrop, this study addresses a critical gap in current literature. While existing systematic reviews aggregate data across ICIs as a class, our work provides the first comprehensive, dual-perspective analysis of camrelizumab-specific ADRs by integrating two complementary approaches: (1) a global pharmacovigilance analysis using the WHO VigiAccess database to capture large-scale real-world ADR signals that reflect post-marketing surveillance patterns; (2) a systematic evaluation of published case reports/case series to detail clinical manifestations, temporal patterns, and management strategies. This dual-source design is deliberately chosen because VigiAccess provides quantitative signal detection across large populations but lacks clinical granularity, while case reports offer rich clinical phenotyping but are subject to publication bias. By synthesizing these complementary data streams, we achieve a more complete safety profile than either source alone. Notably, camrelizumab’s unique association with reactive cutaneous capillary endothelial proliferation (RCCEP),rarely reported with other PD-1 inhibitors,underscores the necessity of drug-specific analyses beyond generic ICI reviews. Ultimately, our findings aim to optimize clinical decision-making for camrelizumab and contribute nuanced insights into the safety of PD-1 inhibitors with distinct ADR profiles.

## 2 Methods

This study was designed as a retrospective pharmacovigilance analysis. Data on ADRs related to camrelizumab were retrieved from the publicly available VigiAccess database of the WHO for the period from June, 2019 to July, 2025. The retrieved data were analyzed to assess the safety profile of camrelizumab. Adverse events (AEs) was systematically classified according to Systemic Organ Classification (SOC), Preferred Terms (PT), and the Medical Dictionary of Regulatory Activities (MedDRA). Download and run MedDRA Desktop Browsers available on MedDRA Web site (https://www.Meddra.org/), import PT and the system will automatically output the SOC mapping table. The demographic data extracted encompasses patient characteristics such as gender and age, reporting schedules, geographical distribution, and the specific organ systems affected by adverse events.This study does not require ethical approval because it used the WHO-VigiAccess database, which is a free open access database.

### 2.1 Descriptive study

#### 2.1.1 Search strategy.

A comprehensive search was conducted in multiple electronic databases, including PubMed and Web of Science, for publications related to camrelizumab from 2019–2025, with the language restricted to English. The search strategy employed was as follows: (((Camrelizumab) OR (SHR-1210))AND (Drug Related Side Effects OR Adverse Reactions)). The inclusion criteria for the articles were: case reports and case series on ADRs induced by camrelizumab, full-text articles in English. The exclusion criteria were: review articles, mechanistic studies, studies with only abstracts available, animal experiments, and duplicate cases.

#### 2.1.2 Explicit quality assessment tool.

The quality of the included case reports was assessed using the standardized CARE (Case report) guidelines checklist for case reports, which includes 13 core items covering title, keywords, abstract, introduction, patient information, clinical findings, timeline, diagnostic assessment, therapeutic interventions, follow-up and outcomes, discussion, patient perspective, and informed consent. Only case reports meeting at least 10 of 13 CARE criteria were included in the final analysis.

#### 2.1.3 Explanation for geographic focus.

Camrelizumab is primarily approved and marketed in China, which consequently results in a predominance of clinical experience and case publications originating from this country. As of 2025, China accounts for >90% of global camrelizumab prescriptions. Therefore, focusing on these cases provides the most relevant and concentrated real-world data currently available for this drug, though we acknowledge this limits generalizability to other populations.

#### 2.1.4 PRISMA flow diagram reference.

**Fig 1:**
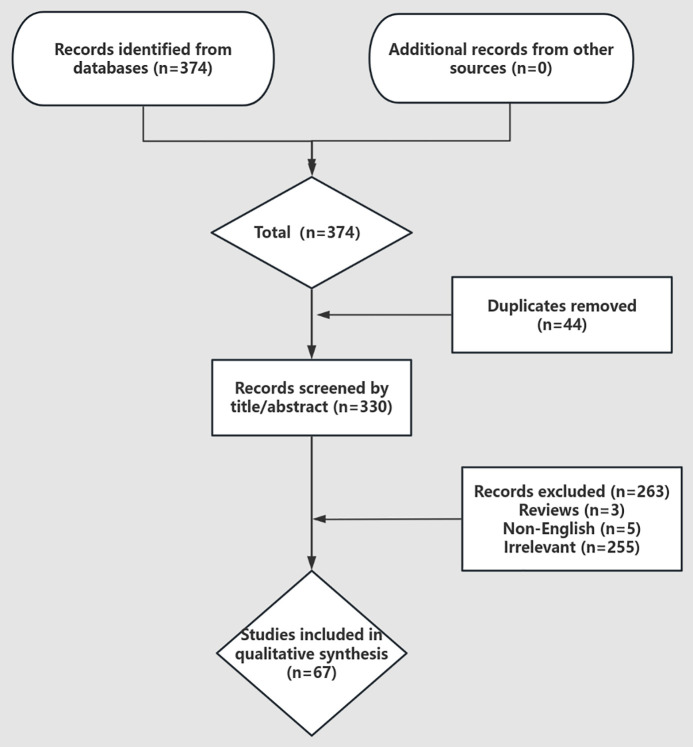
Article selection methodology.

The inclusion process for case and case series studies is shown in Fig 1, which details the identification, screening, eligibility assessment, and inclusion phases.

### 2.2 Clinical data extraction

A structured data extraction form was designed to collect clinical information from the identified articles, including patient demographics (gender, age, primary disease, history of drug use or combination therapy), clinical manifestations, other autoimmune-related ADRs, treatments, and outcomes. The extracted data were summarized and analyzed to provide a comprehensive overview of the ADRs associated with camrelizumab.

### 2.3 Statistical analysis

The extracted data were analyzed using Sigmastat 4.0. Categorical data were presented as counts and percentages, while continuous data were expressed as medians with ranges (minimum and maximum values).

## 3 Results

### 3.1 Data limitations statement

It is important to note that the WHO VigiAccess database provides de-identified individual case safety reports without denominator data (total exposed population) or comparison drug data. Consequently, disproportionality analyses such as Reporting Odds Ratio (ROR), Proportional Reporting Ratio (PRR), or Information Component (IC) could not be performed. The frequencies reported here represent crude counts and proportions among reported cases, not true incidence rates or statistical signals of disproportionate reporting. All associations should be interpreted as descriptive patterns requiring further validation through controlled studies.

### 3.2 Adverse drug reactions of camrelizumab in WHO-VigiAccess

As of July, 2025, the WHO-VigiAccess database recorded a total of 601 ADR reports associated with camrelizumab. The demographic and clinical characteristics of these reports are summarized in Table 1.

**Table 1:** Characteristics of ADR reports of Camrelizumab.

Characteristics	Number of cases(N)	Caseproportion,%
Sex		
female	185	30.78
male	414	68.89
unknow	2	0.33
Age(years)		
12-17	1	0.17
18-44	42	6.99
45-64	315	52.41
65-74	188	31.28
≥65	47	7.82
unknow	8	1.33
Year of reports		
2024	3	0.50
2023	490	81.53
2022	107	17.80
2021	1	0.17
Area of reports		
Americas	3	0.50
Asia	597	99.33
Oceania	1	0.17

Among the 601 ADR reports, 99% (597/601) of the patients were from Asia, and 69% were male. Table 2 shows the five most common adverse reactions of camrelizumab as follows: myelosuppression 105 cases (10.28%), white blood cell count decreased 72 cases (7.05%), rash 58 cases (5.68%). There were 52 cases (5.09%) of thrombocytopenia and 46 cases (4.51%) of neutrophil count decreased.

**Table 2:** Top 30 ADRs of Camrelizumab in the WHO-VigiAccess database.

PTs	Number of cases(N)	proportion,%
Myelosuppression	105	10.28
White blood cell count decreased	72	7.05
Rash	58	5.68
Thrombocytopenia	52	5.09
Neutrophil count decreased	46	4.51
Pruritus	44	4.31
Pyrexia	27	2.64
Hepatic function abnormal	26	2.55
Chest pain	21	2.06
Dyspnoea	19	1.86
Pneumonia	17	1.67
Nausea	16	1.57
Vomiting	15	1.47
Asthenia	14	1.37
Liver injury	14	1.37
Diarrhoea	13	1.27
Haemangioma	13	1.27
Interstitial lung disease	13	1.27
Myocarditis	12	1.18
Hemoglobin decreased	11	1.08
Anaemia	10	0.98
Hyperhidrosis	10	0.98
Chills	9	0.88
Transaminases increased	9	0.88
Telangiectasia	9	0.88
Hypothyroidism	8	0.78
Pancytopenia	7	0.69
Hyperpyrexia	7	0.69
Anaphylactoid reaction	7	0.69
Cough	7	0.69

The most common system organ classes (SOCs) were hematologic disorders (n = 184, 30.62%), skin reactions (n = 121, 20.13%), and general disorders (n = 74, 12.31%). The comprehensive details regarding the SOC are presented in Table 3.

**Table 3:** Distribution of AE signals in each SOC.

SOC	Number of cases (N)	proportion,%
Blood and lymphatic system disorders	184	21.78
Cardiac disorders	31	3.67
Congenital, familial and genetic disorders	1	0.12
Endocrine disorders	18	2.13
Eye disorders	6	0.71
Gastrointestinal disorders	55	6.51
General disorders and administration site conditions	74	8.76
Hepatobiliary disorders	50	5.92
Immune system disorders	13	1.54
Infections and infestations	23	2.72
Injury, poisoning and procedural complications	5	0.59
Investigations	123	14.56
Metabolism and nutrition disorders	18	2.13
Musculoskeletal and connective tissue disorders	12	1.42
Neoplasms benign, malignant and unspecified (incl cysts and polyps)	14	1.66
Nervous system disorders	19	2.25
Psychiatric disorders	5	0.59
Renal and urinary disorders	9	1.07
Reproductive system and breast disorders	1	0.12
Respiratory, thoracic and mediastinal disorders	47	5.56
Skin and subcutaneous tissue disorders	121	14.32
Surgical and medical procedures	1	0.12
Vascular disorders	15	1.78

### 3.3 Descriptive analysis

A total of 67 studies, including 80 case reports, were identified from the literature search. The clinical characteristics of these cases are summarized in Table 4. Among the 80 cases, 58 were male (72.5%) and 22 were female (27.5%), with a median age of 60 years (range 20–84 years). All cases were from China, as the drug is primarily marketed in this country. The most common indications for camrelizumab treatment were non-small cell lung cancer (NSCLC, 16 cases, 20%), nasopharyngeal carcinoma (NPC, 16 cases, 20%), and hepatocellular carcinoma (HCC, 9 cases, 11.25%). The median time to ADR onset was 8 weeks (range 10 minutes–88 weeks), with 16% of the ADRs occurring within 24 hours of the first dose. A total of 68 patients were receiving concomitant medications.

**Table 4:** Basic characteristics of the 80 patients included.

ID	Primary disease	Combination Therapy*	Time to ADR Onset	Reference
1	Non-small cell lung cancer	Yes	3 weeks	[4]
2	Nasopharyngeal carcinoma	Yes	15 minutes after the start of the fifth infusion	[5]
3	Esophageal squamous cell carcinoma	Yes	15 days after 4th cycle	[6]
4	Stage IV left lung adenocarcinoma	Yes	8 days	[7]
5	Acral malignant melanoma	Yes	6 months	[8]
6	Nasopharyngeal squamous cell carcinoma	Yes	18 days after 3rd cycle	[9]
7	Esophageal squamous carcinoma	Yes	1 day after 1st cycle	[9]
8	Cervical esophageal/hypopharyngeal squamous carcinoma	Yes	12 days after 3rd cycle	[9]
9	Non-small cell lung cancer	Yes	1 month	[10]
10	Hepatocellular carcinoma	Yes	3 months	[11]
11	Recurrent nasopharyngeal carcinoma	Yes	4.5 months	[12]
12	Esophageal squamous cell carcinoma	Yes	Immediately	[13]
13	Squamous cell carcinoma of the floor of the mouth	Yes	30 minutes after the 8th camrelizumab infusion	[14]
14	Urethral meatus squamous cell carcinoma	Yes	25 weeks	[15]
15	Lung adenocarcinoma	Yes	5 cycles	[16]
16	Lung adenocarcinoma	Yes	12 cycles	[16]
17	Lung adenocarcinoma	Yes	6 cycles	[16]
18	Gastric adenocarcinoma	Yes	12 cycles	[16]
19	Cervical cancer	Yes	4 cycles	[16]
20	Esophageal squamous cell carcinoma	Yes	4 cycles	[16]
21	Gastric adenocarcinoma	Yes	5th cycle	[17]
22	Metastatic tongue squamous cell carcinoma	No	1 week after 2nd cycle	[18]
23	Non-small cell lung cancer	No	10 days after 2nd cycle	[18]
24	Cervical carcinoma	Yes	1 week after 1st cycle	[19]
25	Nasopharyngeal carcinoma	Yes	3 months	[20]
26	Esophageal squamous cell carcinoma	Yes	8 weeks	[21]
27	Esophageal cancer with hepatic metastases	Yes	6 weeks	[22]
28	Metastatic esophageal squamous cell carcinoma	No	1 year	[23]
29	Hepatocellular carcinoma	Yes	18 days	[24]
30	Stage IV non-small cell lung cancer	Yes	2 weeks	[25]
31	Stage IV hypopharynx cancer	Yes	32 weeks	[26]
32	HBV-related hepatocellular carcinoma	Yes	3 weeks	[27]
33	Non-squamous NSCLC	Yes	6 months	[28]
34	Non-squamous NSCLC	Yes	22 months	[28]
35	Advanced NSCLC	Yes	After 2 cycles	[29]
36	Advanced hepatocellular carcinoma	Yes	3rd cycle	[30]
37	Lung cancer	Yes	2nd cycle	[31]
38	Hepatocellular carcinoma	Yes	21 days	[32]
39	Endometrial serous adenocarcinoma	Yes	1 month	[33]
40	Esophageal squamous cell carcinoma	Yes	6 weeks	[34]
41	Small-cell lung cancer	Yes	8 months	[35]
42	Lung adenocarcinoma with brain and bone metastases	Yes	17 days	[36]
43	Hepatocellular carcinoma	Yes	3 cycles	[37]
44	Gastric adenocarcinoma	Yes	1 month	[38]
45	Rectal endocrine tumor	Yes	10 days after two cycles	[39]
46	Oesophageal squamous cancer	Yes	7 doses of camrelizumab	[40]
47	Small cell lung cancer	Yes	10th dose of camrelizumab	[41]
48	Gastric antrum cancer	Yes	10 months	[42]
49	Advanced non-small-cell lung cancer	Yes	11 months	[43]
50	De novo metastatic nasopharyngeal carcinoma	Yes	16 weeks	[44]
51	Non-small-cell lung carcinoma	No	22 weeks	[45]
52	Hepatocellular carcinoma	Yes	20 weeks	[45]
53	Squamous cell carcinoma of upper lobe of left lung with mediastinal lymph node and liver metastases	Yes	4 days after 5th cycle	[46]
54	Poorly differentiated adenocarcinoma of middle-lower esophagus with lymph node metastasis	Yes	10th cycle	[47]
55	Stage IV Lung adenocarcinoma	Yes	3rd Camrelizumab cycle	[48]
56	Stage IV non-small cell lung cancer	Yes	6 weeks	[49]
57	Metastatic thymoma	No	11 days	[50]
58	Gastric cancer	Yes	6 months	[51]
59	Esophageal squamous cell carcinoma with liver metastasis	No	second dose of camrelizumab	[52]
60	Liver cancer with lung metastasis	Yes	second dose of camrelizumab	[53]
61	Advanced non–small-cell lungcancer	No	three months	[54]
62	Advanced gallbladder carcinoma	Yes	3 days	[55]
63	Esophageal squamous cell carcinoma	Yes	3 weeks	[56]
64	Non-small cell lung cancer	No	first injection	[57]
65	Soft tissue sarcoma	Yes	11 days	[58]
66	NSCLC	Yes	20 days	[59]
67	Liver cancer	Yes	1 week	[60]
68	Lung cancer	Yes	3 weeks	[60]
69	Esophageal cancer with lymph node metastasis	Yes	5 weeks	[61]
70	Advanced esophageal squamous cell carcinoma	Yes	12 days after 3rd cycle	[62]
71	Esophageal cancer	Yes	20 days	[63]
72	Stage IIIB NSCLC	Yes	10 weeks	[64]
73	Stage IVA squamous NSCLC	Yes	6 weeks	[64]
74	Gastric adenocarcinoma	Yes	15 weeks	[65]
75	NSCLC	No	112 days	[66]
76	Metastatic gallbladder adenocarcinoma	Yes	41 days	[67]
77	Lung cancer	Yes	6 months	[68]
78	Stage IV lung adenocarcinoma	Yes	6 weeks	[69]
79	Stage IVa Hodgkin’s lymphoma	No	1 month	[70]
80	Stage IV lung adenocarcinoma	No	2 weeks	[70]

*Combination Therapy: Yes = combination with chemotherapy/targeted therapy; No = camrelizumab monotherapy.

**Note:** Detailed treatment regimens (drug dosages, cycles, radiation details) are provided in S1 Supplementary.

### 3.4 Clinical manifestations, laboratory examination, management and treatment outcomes

Table 5 summarizes the clinical manifestations of adverse reactions in 80 patients: skin toxicity (45 cases, 56.3%); RCCEP (32 cases), SJS/TEN (5 cases); Hematological toxicity (38 cases, 47.5%): bone marrow suppression (22 cases), thrombocytopenia (16 cases); Cardiotoxicity (13 cases, 16.25%), mainly myocarditis; Others: Immune hepatitis (6 cases), thyroid dysfunction (5 cases). Table 5 also summarizes the prognosis of patients after taking corresponding treatments for adverse reactions: The discontinuation rate of camrelizumab: 92.5% (74/80); Glucocorticoid utilization rate: 85% (68/80), median starting dose methylprednisolone 1 mg/kg; Immunoglobulin utilization rate: 13.75% (11/80). After corresponding treatment or drug withdrawal, the improvement rate of patients was 45% (44/80). Among the deaths (7 cases, 8.8%), the causes of death included myocarditis (3 cases), liver failure (2 cases), and septic shock (2 cases).

**Table 5:** Summary of Clinical Manifestations and Prognosis of ADRs in 80 Patients.

Category	Specific ADRs	Number of Cases (N)	Proportion (%)
**Clinical Manifestations**			
Skin toxicity	Overall skin toxicity	45	56.3
	Reactive cutaneous capillary endothelial proliferation (RCCEP)	32	
	Stevens-Johnson syndrome (SJS)/Toxic epidermal necrolysis (TEN)	5	
Hematological toxicity	Overall hematological toxicity	38	47.5
	Bone marrow suppression	22	
	Thrombocytopenia	16	
Cardiotoxicity	Overall cardiotoxicity (mainly myocarditis)	13	16.25
Others	Immune hepatitis	6	
	Thyroid dysfunction	5	
**Prognosis After Treatment**			
Camrelizumab discontinuation rate		74	92.5
Glucocorticoid utilization rate	median starting dose: methylprednisolone 1 mg/kg	68	85
Immunoglobulin utilization rate		11	13.75
Improvement rate	after treatment/drug withdrawal	44	45
Deaths	Total deaths	7	8.8
	Myocarditis	3	
	Liver failure	2	
	Septic shock	2	

**Note:** Detailed laboratory values (specific troponin levels, CBC results, liver function tests), complete imaging findings, and specific drug dosing regimens are provided in S2 Supplementary.

## 4 Discussion

### 4.1 Core safety concerns

Skin toxicity in this study, 56.3% of the cases presented with skin ADR, which was higher than that of other ICIs skin immune-related adverse events (25.1%) [71]. The incidence of RCCEP, a camrelizumab specific reaction, was 40% (32/80), possibly due to the reactivation of the immune response caused by camrelizumab. It disrupts the balance between angiogenic growth factors and anti-angiogenic growth factors, causing abnormal proliferation of skin capillary endothelial cells and ultimately leading to the occurrence of RCCEP, which is an immune stress response.[72]

Hematological toxicity: The incidence of bone marrow suppression caused by WHO-VigiAccess accounted for 10.28% of all adverse reactions, ranking first and accounting for 47.5% of the studies included in the literature, while the incidence of thrombocytopenia was 20%. Hematopoietic stem cells are the origin of blood cell production and can differentiate into various types of blood cells. ICI is often used in combination with chemotherapy or targeted therapy, and the overlapping mechanism may directly or indirectly damage hematopoietic stem cells, exacerbating hematological toxicity.[73] Other chemotherapy drugs can independently activate the immune system. When used in combination with ICI, this dual activation may lead to excessive proliferation of immune cells, thereby attacking hematopoietic stem cells, inhibiting bone marrow hematopoiesis, reducing blood cell production, and increasing hematological toxicity. Therefore, when camrelizumab is used in combination with chemotherapy drugs, regular blood routine monitoring is required.

Serious late-onset events: Late-onset serious ADRs, such as myocarditis (median onset time: 9 weeks) and toxic epidermal necrolysis (TEN) (median onset time: 5.5 weeks), pose significant challenges in the management of patients treated with camrelizumab. These ADRs are associated with high mortality rates (8.8%), emphasizing the need for early detection and intervention.

Early identification and glucocorticoid intervention are key management strategies. The treatment of myocarditis requires adequate rest and vitamin supplementation. During the acute stage, more rest is needed. In the later stage, rest for 1–3 months. Physical labor and strenuous exercise should be avoided within one year. Patients need to regularly monitor their myocardial enzyme spectrum and electrocardiogram to be vigilant about arrhythmia. TEN treatment should be discontinued promptly, with skin care and supportive treatment provided to prevent infection.

### 4.2 Risk factors

Combination therapy: The majority (85%) of the cases in this study involved the use of camrelizumab in combination with chemotherapy or targeted drugs (e.g., apatinib). This combination therapy approach, while potentially enhancing the therapeutic efficacy, also appears to increase the risk of hepatotoxicity and bleeding through synergistic toxicity. [74]The concurrent use of multiple therapeutic agents can lead to complex pharmacodynamic interactions, which may exacerbate the overall toxicity profile. Therefore, careful consideration should be given to the potential risks and benefits when combining camrelizumab with other therapeutic agents. Close monitoring of liver function tests and coagulation parameters is essential in patients receiving combination therapy to detect and manage any signs of hepatotoxicity or bleeding promptly.

Gender and age: The findings of this study suggest that males (72.5%) and elderly patients (≥75 years) are more susceptible to cardiac toxicity associated with camrelizumab. This observation is consistent with the trends observed for other immune checkpoint inhibitors (ICIs).[75]The increased vulnerability of elderly patients may be attributed to age-related physiological changes, such as decreased organ function and altered pharmacokinetics, which can affect the metabolism and clearance of the drug. Additionally, elderly patients often have a higher prevalence of comorbid conditions, which may further complicate their clinical management. Gender differences in the incidence of cardiac toxicity may be related to biological and hormonal factors that influence the immune response and cardiovascular function. These findings highlight the need for tailored management strategies based on patient demographics to optimize the safety and efficacy of camrelizumab therapy.

### 4.3 Management strategies

Effective management of camrelizumab-associated adverse drug reactions (ADRs) requires a structured, evidence-based approach prioritizing early recognition, severity-stratified intervention, and outcome optimization. Below, we outline key strategies linked to specific ADR types, emphasizing their proven efficacy in mitigating morbidity and mortality.

Graded intervention: A graded approach to managing ADRs is recommended to ensure appropriate and timely intervention based on the severity of the reaction. For grade 1–2 ADRs, local treatment or temporary discontinuation of the drug may be sufficient to allow the symptoms to resolve. However, for grade 3–4 ADRs, more aggressive management is warranted. Permanent discontinuation of camrelizumab and initiation of corticosteroid therapy (e.g., methylprednisolone 1–2 mg/kg) are advised to control the immune-mediated inflammation effectively.[76] The graded intervention strategy aims to balance the need for effective ADR management with the potential benefits of continued therapy in patients with lower-grade reactions.

Special event management: For myocarditis, a life-threatening ADR with reported mortality up to 50% if untreated. Early combination therapy (within 72 hours of diagnosis) with high-dose corticosteroids (methylprednisolone 1–2 mg/kg/day) + intravenous immunoglobulin (IVIG; 2 g/kg divided over 2–5 days) is strongly recommended. Retrospective cohort studies demonstrate this regimen reduces mortality by 30–40% compared to steroids alone, attributed to synergistic suppression of myocardial autoimmunity and stabilization of left ventricular ejection fraction (LVEF). [9]In cases of TEN, a devastating cutaneous ADR with historical mortality of 30–50%. A multidisciplinary team approach (dermatology, ICU, ophthalmology, nutrition support) is indispensable. First-line therapy combines: (1) high-dose corticosteroids (pulse methylprednisolone 10–15 mg/kg/day for 3 days, then taper), (2) IVIG (3–4 mg/kg/day for 3–4 days) to inhibit Fas-FasL-mediated keratinocyte apoptosis, and (3) plasma exchange (for extensive epidermal detachment >30% BSA). Supportive care (wound care, fluid/electrolyte management, sepsis prophylaxis) reduces secondary complications. Case series report a 20–25% improvement in 28-day survival with this protocol compared to historical controls. For RCCEP, unique to camrelizumab, this benign but cosmetically concerning ADR is managed with local therapy (intralesional corticosteroids, laser ablation) and dose modification (temporary hold for grade 3). Severe cases (50% body surface area) may require permanent discontinuation, though most respond to conservative measures without impacting survival.

### 4.4 Clinical recommendations

Based on our comprehensive analysis of camrelizumab-associated ADRs, we propose the following evidence-based recommendations to optimize clinical management:

#### 4.4.1 Proactive, time-window-guided monitoring.

(1) Baseline assessment:Cardiac enzymes (troponin, CK-MB), liver function tests, thyroid function, and complete blood count should be obtained before initiation of camrelizumab.
(2) Intensive monitoring:Heightened vigilance for ADRs is recommended during the first 8 weeks of treatment, when the majority of events occur.
(3) Long-term surveillance: For patients on extended therapy, periodic monitoring of cardiac and endocrine function is advised given the potential for delayed-onset irAEs.

#### 4.4.2 Patient education and reporting.

Patients should be educated on common ADRs and instructed to report any new or worsening symptoms promptly.Healthcare providers should maintain high index of suspicion for irAEs, particularly in patients with pre-existing autoimmune conditions.

### 4.5 Limitations and generalizability considerations

A notable limitation of this study lies in the geographical concentration of adverse drug reaction (ADR) reports within the WHO VigiAccess database. As identified, approximately 99% of camrelizumab-related reports in VigiAccess originated from Asian countries, with a predominant contribution from China, where camrelizumab was first approved and has been widely adopted in clinical practice since 2019. This regional skew raises concerns regarding the global generalizability of our pharmacovigilance findings. The observed ADR profile may therefore primarily reflect the safety experience of Asian patient populations, particularly those in East Asia, and may not fully represent the safety profile in other ethnic groups or healthcare contexts.

Several factors likely contribute to this geographical concentration: (1) camrelizumab received its first regulatory approval in China in May 2019 and was primarily marketed in Asia during the study period; (2) reporting practices and pharmacovigilance infrastructure vary significantly across regions, potentially leading to under-reporting from non-Asian countries; (3) genetic polymorphisms in drug metabolism and immune response genes may influence ADR susceptibility, though our data cannot directly test this hypothesis.

Furthermore, the VigiAccess database lacks denominator data (total number of patients exposed to camrelizumab), precluding calculation of true incidence rates and limiting our analysis to descriptive frequencies. As a passive reporting system, VigiAccess is also subject to inherent limitations including reporting bias, incomplete data fields, and inability to establish causal relationships between camrelizumab and reported events. The observed associations should be interpreted as pharmacovigilance signals requiring further validation rather than confirmed adverse reactions.

While this limitation tempers broad extrapolation, it is important to contextualize the findings within Asia’s role as a key region for camrelizumab use. The data still provide valuable insights into the safety profile of the drug in its most extensively studied population. However, future research should prioritize multi-center, cross-ethnic studies integrating additional global databases (e.g., FAERS, EudraVigilance) and prospective observational cohorts to validate these observations and clarify potential racial/ethnic variations in camrelizumab-associated ADRs. Such efforts will be critical to refining risk stratification and personalized safety monitoring strategies worldwide.

## 5 Conclusion

This study provides a comprehensive analysis of camrelizumab-related adverse events using both VigiAccess data and a case-based literature review. The dual-source approach enhances our understanding of the drug’s safety profile. Key safety concerns include hematological disorders, skin reactions, and serious events such as myocarditis and toxic epidermal necrolysis. Monitoring priorities should focus on the first 8 weeks of treatment, when most ADRs occur. Future research should address the limitations of the current study, including geographical reporting bias and the need for disproportionality analyses. Additionally, further investigation into the management of camrelizumab-related ADRs and their long-term outcomes is warranted.

## Supporting information

S1 Supplementary Detailed treatment plan and the time of the first adverse reaction occurrence. (DOC)

S2 Supplementary Detailed laboratory values,complete imaging findings, and specific drug dosing regimens. (DOC)
