# Supplementary material for: Safety profile of camrelizumab: An analysis based on literature and database review
Source: PLoS One. 2026 Jul 23;21(7):e0354252. doi: 10.1371/journal.pone.0354252 (PMC13395421; doi:10.1371/journal.pone.0354252)
Supplement: S1 Supplementary — (DOC) [file pone.0354252.s001.doc]

| **ID** | **History of drug use or combination therapy** | **The first onset time after initiation of treatment** | **Reference** |
| --- | --- | --- | --- |
| 1 | Camrelizumab (200 mg, Day 1) combined with chemotherapy: Paclitaxel (210 mg, Day 1) and Carboplatin (340 mg, Day 1), administered every 3 weeks for three cycles. | After the first course of treatment (approximately 3 weeks post-initiation) | (1) |
| 2 | Camrelizumab (200 mg every 3 weeks) combined with nab-paclitaxel and nedaplatin for the first four cycles (tolerated well). During the fifth cycle, camrelizumab infusion caused anaphylaxis. | 15 minutes after the start of the fifth infusion of camrelizumab | (2) |
| 3 | Camrelizumab + Paclitaxel + Carboplatin (2 cycles) + 30 sessions esophageal radiotherapy,Camrelizumab monotherapy (200mg IV q3w, cycles 3-4) | 15 days after 4th cycle of camrelizumab monotherapy | (3) |
| 4 | Self-administered gefitinib,Camrelizumab monotherapy,Intrapleural cisplatin | 8 days after camrelizumab initiation | (4) |
| 5 | Camrelizumab (200mg IV q3w) + Apatinib (250mg daily),Apatinib discontinued 72 days pre-admission due to drug-induced liver injury | 6 months after camrelizumab initiation (symptoms began >40 days before admission) | (5) |
| 6 | Cisplatin + Gemcitabine + Camrelizumab (200mg IV q3w) | 18 days after 3rd cycle | (6) |
| 7 | Albumin-paclitaxel + Cisplatin + Camrelizumab (200mg IV q3w),1 cycle | 1 day after 1st cycle | (6) |
| 8 | Cisplatin + Albumin-paclitaxel + Camrelizumab (200mg IV q3w) | 12 days after 3rd cycle | (6) |
| 9 | Camrelizumab (200mg IV),Paclitaxel (albumin-bound, 400mg IV),Carboplatin (500mg IV) 3 cycles | 1 month after immunotherapy initiation | (7) |
| 10 | Camrelizumab (200mg IV q3w) + Lenvatinib (8mg daily),Additional TACE + right portal vein embolization | 3 months after camrelizumab initiation | (8) |
| 11 | Albumin-paclitaxel (230 mg/m^2^) + lobaplatin (30 mg/m^2^) + camrelizumab (200 mg) q3w for 6 cycles, Camrelizumab monotherapy for 7th-8th cycles | After 6th cycle of camrelizumab (approx. 4.5 months post-initiation) | (9) |
| 12 | Adjuvant chemotherapy (docetaxel + lobaplatin ×2 cycles),Camrelizumab (200mg) + cisplatin + 5-fluorouracil, Intensity-modulated radiotherapy (54 Gy/28 fractions) to cervical lymph nodes/gastroesophageal anastomosis | Immediately after radiotherapy + camrelizumab initiation | (10) |
| 13 | Camrelizumab (200mg q3w) + Albumin-bound paclitaxel (400mg) + Cisplatin (60mg D1-2),Albumin-bound paclitaxel + Cisplatin,Camrelizumab + Albumin-bound paclitaxel | 30 minutes after the 8th camrelizumab infusion (Cycle 9) | (11) |
| 14 | Camrelizumab (200mg q3w) + Anlotinib (12mg/day) | Hypothyroidism: 25 weeks post-initiation T1DM + Adrenal insufficiency: 45 weeks post-initiation | (12) |
| 15 | Pemetrexed + Nedaplatin + Endostatin (4 cycles),Pemetrexed + Carboplatin + Bevacizumab (6 cycles),Nab-paclitaxel + Bevacizumab + Camrelizumab (8 cycles) | 5 cycles after camrelizumab initiation | (13) |
| 16 | Gefitinib + Bevacizumab,Osimertinib + Bevacizumab,Pemetrexed + Carboplatin + Bevacizumab,Nab-paclitaxel + Bevacizumab,Camrelizumab + Bevacizumab (16 cycles) | 12 cycles after camrelizumab initiation | (13) |
| 17 | Pemetrexed + Carboplatin,Nab-paclitaxel + Bevacizumab + Camrelizumab | 6 cycles after camrelizumab initiation | (13) |
| 18 | SOX + Trastuzumab + Camrelizumab (12 cycles) | 12 cycles after camrelizumab initiation | (13) |
| 19 | Docetaxel + Carboplatin + Endostatin,Nab-paclitaxel + Cisplatin,Nab-paclitaxel + Camrelizumab | 4 cycles after camrelizumab initiation | (13) |
| 20 | TP + Nimotuzumab,Nab-paclitaxel + Camrelizumab + Nimotuzumab | 4 cycles after camrelizumab initiation | (13) |
| 21 | ​ Radical distal gastrectomy,Adjuvant SOX (oxaliplatin + tegafur/gimeracil/oteracil,8 cycles),Radiotherapy,Trastuzumab + albumin-bound paclitaxel (8 cycles, PR → PD),Trastuzumab + camrelizumab + apatinib (13 cycles, PR) | Grade II diarrhea (after 5th cycle),Progressive rash/pruritus (each cycle),Grade II oral ulcer (after 13th cycle) | (14) |
| 22 | Camrelizumab monotherapy | 1 week after 2nd cycle | (15) |
| 23 | Camrelizumab monotherapy | 10 days after 2nd cycle | (15) |
| 24 | Camrelizumab + chemotherapy (paclitaxel/cisplatin) + radiotherapy | 1 week after 1st cycle | (16) |
| 25 | Camrelizumab + chemotherapy (capecitabine) | 3 months after initiation | (17) |
| 26 | Camrelizumab + paclitaxel | 8 weeks after initiation | (18) |
| 27 | Camrelizumab with paclitaxel (albumin-bound) and carboplatin | After 2 cycles (~6 weeks) | (19) |
| 28 | Camrelizumab monotherapy | After 1 year (oral erosion over 2 months) | (20) |
| 29 | Lenvatinib (12 mg daily) + Camrelizumab (200 mg IV every 3 weeks) | 18 days after first Camrelizumab injection | (21) |
| 30 | Camrelizumab monotherapy (200 mg IV every 2 weeks) | After 2 weeks of initial Camrelizumab treatment | (22) |
| 31 | Camrelizumab (200 mg IV) + albumin paclitaxel (260 mg/m²) + cisplatin (75 mg/m²) every 3 weeks (4 cycles),Weekly cisplatin (40 mg/m²) + radiotherapy (67.84 Gy to laryngopharynx/metastatic nodes; 58.24 Gy to esophagus),Camrelizumab monotherapy (200 mg every 3 weeks, 2 cycles) | 32 weeks after first Camrelizumab dose (8 months post-induction, 3 months post-chemoradiation) | (23) |
| 32 | TACE + Camrelizumab | 3 weeks | (24) |
| 33 | Camrelizumab + Bevacizumab (2nd-line) | 6 months | (25) |
| 34 | Camrelizumab + Pemetrexed/Carboplatin,Camrelizumab maintenance | 22 months | (25) |
| 35 | Camrelizumab 200 mg q3w + nedaplatin + pemetrexed + Endostar/bevacizumab | After 2 cycles (inferred from disease course) | (26) |
| 36 | Camrelizumab (200 mg q3w),Apatinib (250 mg daily),TACE ×4, Microwave ablation | 3rd cycle of camrelizumab | (27) |
| 37 | Pemetrexed + Carboplatin (chemotherapy),Camrelizumab (immunotherapy),Thalidomide (50 mg AM/100 mg PM) | 2nd cycle of camrelizumab + chemotherapy | (28) |
| 38 | Camrelizumab (200mg ×4 courses),Sorafenib (400mg bid,6 months),Chemotherapy:(pirarubicin/fluorouracil/oxaliplatin/gemcitabine/cisplatin) | 21 days after last camrelizumab dose | (29) |
| 39 | Paclitaxel + cisplatin (adjuvant),Albumin paclitaxel + carboplatin,Camrelizumab (PD-1 inhibitor) | 1 month after camrelizumab initiation | (30) |
| 40 | Camrelizumab (200 mg every 3 weeks) combined with chemotherapy (reduced-dose Tc regimen) and radiotherapy | After 6 weeks of treatment | (31) |
| 41 | Camrelizumab (200 mg every 3 weeks) combined with anlotinib (8 mg daily for 2 weeks on/1 week off) | After approximately 8 months of treatment | (32) |
| 42 | 1 cycle of pemetrexed + carboplatin chemotherapy,After PD-L1 testing: 1 cycle of camrelizumab (200 mg) + pemetrexed + carboplatin | 17 days after camrelizumab initiation | (33) |
| 43 | Transcatheter arterial chemoembolization (TACE) + sorafenib (400 mg twice daily),Camrelizumab (200 mg every 21 days) for 3 cycles | After 3 cycles of camrelizumab (approximately 63 days post-initiation, as each cycle is 21 days) | (34) |
| 44 | Camrelizumab combined with oxaliplatin and capecitabine | ~1 month | (35) |
| 45 | Camrelizumab combined with sorafenib | 10 days after two cycles | (36) |
| 46 | Radical surgery,Camrelizumab 200 mg every 2 weeks (second-line therapy) + radiation (30Gy/10F/3Gy to bone lesion) | After 7 doses of camrelizumab | (37) |
| 47 | Chemotherapy (IP: irinotecan + cis-platinum; EN: irinotecan + nedaplatin) + prophylactic cranial irradiation + radiation to iliac region,Camrelizumab 200 mg every 3 weeks | After 10th dose of camrelizumab (vision decrease noticed 6 days after dose) | (38) |
| 48 | ​​Chemotherapy: Nab-paclitaxel (d1, d15),Targeted therapy: Trastuzumab (d1, d15),Immunotherapy: Camrelizumab (d1, d15; 10 cycles) | 10 months | (39) |
| 49 | Carboplatin + nab-paclitaxel + camrelizumab,camrelizumab monotherapy | Approximately 11 months | (40) |
| 50 | Camrelizumab + Chemotherapy (gemcitabine/cisplatin),Radiotherapy + Nimotuzumab | 16 weeks (after chemoradiotherapy) | (41) |
| 51 | Camrelizumab monotherapy | 22 weeks (after 11 cycles) | (42) |
| 52 | Camrelizumab + Apatinib | 20 weeks (after 10 cycles) | (42) |
| 53 | Chemotherapy: Paclitaxel (albumin-bound) + Cisplatin Immunotherapy: Camrelizumab (200 mg, every 3 weeks) | 4 days after 5th cycle of camrelizumab | (43) |
| 54 | Chemotherapy: Paclitaxel/Carboplatin,Cisplatin/Nedaplatin Immunotherapy: Camrelizumab (10 cycles) | Acute onset after 10th cycle of camrelizumab | (44) |
| 55 | Carboplatin + Pemetrexed + Camrelizumab | After 3rd Camrelizumab cycle (Acute Mastitis) | (45) |
| 56 | Pemetrexed + Carboplatin + Camrelizumab | After 6 weeks (Vitiligo aggravation) | (46) |
| 57 | Camrelizumab monotherapy (200 mg intravenously every 3 weeks) | 11 days after the first dose | (47) |
| 58 | Camrelizumab (200 mg intravenously every 3 weeks) combined with apatinib mesylate tablets (850 mg/day) | After 6 months (9 cycles) of camrelizumab therapy | (48) |
| 59 | Camrelizumab monotherapy (200 mg every 3 weeks) | After second dose of camrelizumab, within 10 minutes | (49) |
| 60 | Camrelizumab (200 mg every 3 weeks) combined with regorafenib (160 mg daily) and entecavir (0.5 mg daily) | After second dose of camrelizumab (specific time not specified; symptoms noted during follow-up) | (50) |
| 61 | Camrelizumab (200 mg, ivgtt, q3w) | three months | (51) |
| 62 | Camrelizumab (200 mg every 3 weeks) + apatinib (250 mg daily) | 3 days after starting combination therapy | (52) |
| 63 | Camrelizumab (200 mg per injection) combined with chemotherapy (paclitaxel 135 mg/m² + cisplatin 75 mg/m²) | 3 weeks after first dose of camrelizumab | (53) |
| 64 | SHR-1210 (Camrelizumab) injections | Skin reactive capillary hemangiomas: After first injection | (54) |
| 65 | Anlotinib 200 mg qd po + camrelizumab 200 mg q21d for two cycles,Pazopanib 800 mg qd po + camrelizumab for one cycle. | 11 days | (55) |
| 66 | Pemetrexed + Bevacizumab + Camrelizumab | 20 days | (56) |
| 67 | Apatinib + Camrelizumab | 1 week | (57) |
| 68 | Paclitaxel + Nedaplatin + Camrelizumab | 3 weeks | (57) |
| 69 | Capecitabine + camrelizumab | 5 weeks | (58) |
| 70 | Tislelizumab/placebo + capecitabine + cisplatin,Nimotuzumab + docetaxel,Camrelizumab (200 mg) + docetaxel (140 mg)​ | 12 days after 3rd cycle | (59) |
| 71 | Camrelizumab (200 mg, administered twice with a 2-week interval),Capecitabine (1.5 g orally twice daily for 14 days) | Approximately 20 days after camrelizumab administration | (60) |
| 72 | Adjuvant chemo (pemetrexed + cisplatin),Radiotherapy (50 Gy),Pemetrexed/gefitinib/osimertinib,MWA+camrelizumab​​ (200mg q2w) | 10 weeks (after 5 cycles of camrelizumab) | (61) |
| 73 | MWA,Camrelizumab(200mg q2w),Docetaxel + cisplatin (disease progression) | 6 weeks (after 3 cycles of camrelizumab) | (61) |
| 74 | Camrelizumab (200 mg every 3 weeks),Systemic chemotherapy: Paclitaxel liposome (intravenous and intraperitoneal), Weikangda (oral Tegafur/Gimeracil/Oteracil Potassium) | After the fifth administration of camrelizumab (approximately 15 weeks post-initiation, based on 3-week cycles) | (62) |
| 75 | Camrelizumab | 112 (after 8th dose) | (63) |
| 76 | Camrelizumab + Gemcitabine | 41 (asymptomatic) | (64) |
| 77 | Camrelizumab (7 months),Nifedipine (1 month) | >6 months after Camrelizumab initiation | (65) |
| 78 | Camrelizumab+pemetrexed/carboplatin (6 cycles),Camrelizumab+pemetrexed (maintenance) | 6 weeks after Camrelizumab initiation | (66) |
| 79 | Camrelizumab | 1 month | (67) |
| 80 | Camrelizumab | 2 weeks | (67) |

**S 1 Detailed treatment plan and the time of the first adverse reaction occurrence**

**References:**

1. Bao H, Zhang J, Luo X, Song X, Li J, Mao N, et al. Case Report: Subacute cutaneous lupus erythematosus induced by the anti-PD-1 antibody camrelizumab combined with chemotherapy. Front Immunol. 2025;16:1539373. http://doi.org/10.3389/fimmu.2025.1539373

2. Song P, Jin Y, Dai L, Fang L, Tan Y. A case of camrelizumab-induced anaphylaxis and successful rechallenge: a case report and literature review. Front Oncol. 2025;15:1537205. http://doi.org/10.3389/fonc.2025.1537205

3. Jiang YJ, Wu L, Yang X, Pu Y, Ning BJ, Peng N, et al. Dermatitis bullosa caused by the immune checkpoint inhibitor camrelizumab: A case report. World J Clin Cases. 2025;13(8):97677. http://doi.org/10.12998/wjcc.v13.i8.97677

4. Sun M, Zhou H, Zang D, Liu CG, Chen J. Camrelizumab-induced immune-related toxic epidermal necrolysis in lung adenocarcinoma: a case report and literature review. Front Oncol. 2024;14:1417936. http://doi.org/10.3389/fonc.2024.1417936

5. Gu Y, Yi L, Zou X, Guo L, Wu G, Zhao J. Refractory hypokalemia and metabolic acidosis induced by undifferentiated connective tissue disease secondary to immune checkpoint inhibitors: a case report and literature review. Front Oncol. 2024;14:1442605. http://doi.org/10.3389/fonc.2024.1442605

6. Ji W, Wei Q, Tang Z, Zhang W. Three Cases of Immune Myocarditis Associated with Camrelizumab Use. Case Rep Oncol. 2024;17(1):1034-41. http://doi.org/10.1159/000540891

7. Wang YY, Song JJ. A case report of the diagnosis and treatment of immune checkpoint inhibitor-related encephalitis induced by camrelizumab. AME Case Rep. 2024;8:101. http://doi.org/10.21037/acr-24-58

8. Wan DL, Hu C, Ke QH, Zhou T, Ruan L, Tang H, et al. Corticosteroid-dependent immune checkpoint inhibitor-induced enterocolitis treated with vedolizumab: a case report. J Gastrointest Oncol. 2024;15(4):1948-56. http://doi.org/10.21037/jgo-24-222

9. Liu Z, Du X, Deng M, Chen Y, Wu S. Successful replacement for recurrent nasopharyngeal carcinoma with cholecystitis induced by PD-1 antibody: a case report. Immunotherapy-Uk. 2024;16(14-15):949-53. http://doi.org/10.1080/1750743X.2024.2382670

10. Wang H, Li Y, Qiu M, Wang J. Thrombocytopenia and hyperprogression after radiotherapy and camrelizumab treatment in an esophageal cancer patient with increased JAK2 gene copies: a case report. Front Oncol. 2024;14:1283428. http://doi.org/10.3389/fonc.2024.1283428

11. Hu J, Fan J, Qu S, He X, Liu D, Wang Y, et al. Camrelizumab-induced anaphylactic reaction: a case report and literature review. J Chemotherapy. 2025;37(4):376-82. http://doi.org/10.1080/1120009X.2024.2372525

12. Pan Q, Li P. Challenges in autoimmune polyendocrine syndrome type 2 with the full triad induced by anti-programmed cell death 1: a case report and review of the literature. Front Immunol. 2024;15:1366335. http://doi.org/10.3389/fimmu.2024.1366335

13. Zhang C, Wang G, Liu N, Li T, Zhu J, Hou H. Case report: A rare immune-related adverse effect: hepatic cavernous hemangioma induced by camrelizumab. Front Immunol. 2024;15:1387465. http://doi.org/10.3389/fimmu.2024.1387465

14. Ma X, Xue L, Ou K, Liu X, Chen J, Gao L, et al. Significant effect of posterior line treatment of HER2 positive advanced gastric cancer: A case report. Heliyon. 2024;10(7):e28923. http://doi.org/10.1016/j.heliyon.2024.e28923

15. Ge L, Chen W, Zhang G, Wei F. Camrelizumab-induced immune-related adverse events: Two case reports and literature review. Asian J Surg. 2024;47(6):2733-4. http://doi.org/10.1016/j.asjsur.2024.03.120

16. Zhou X, Yan X, Wu Y. Reactive cutaneous capillary endothelial proliferations of the eyelids induced by camrelizumab: A case report. Biomed Rep. 2024;20(3):53. http://doi.org/10.3892/br.2024.1743

17. Lin Y, Lin Y, Zhong X, Chen Q, Tang S, Chen J. A case report and literature review on reactive cutaneous capillary endothelial proliferation induced by camrelizumab in a nasopharyngeal carcinoma patient. Front Oncol. 2023;13:1280208. http://doi.org/10.3389/fonc.2023.1280208

18. Xie RX, Xue YB, Ci XY, Zhang MJ. Immune checkpoint inhibitor induced colitis and arthritis: A case report. Medicine. 2023;102(49):e36334. http://doi.org/10.1097/MD.0000000000036334

19. Long HD, Du YP, Wang LY, Liu GC, Liang SX, Zeng ZH, et al. Successful management of camrelizumab-induced immune-checkpoint-inhibitors-related myocarditis. J Oncol Pharm Pract. 2024;30(3):597-604. http://doi.org/10.1177/10781552231216104

20. Wang X, Fu T, Sun W. Camrelizumab-induced oral lichenoid reaction with subepithelial CD4+ T-cell infiltration. J Oncol Pharm Pract. 2024;30(1):228-34. http://doi.org/10.1177/10781552231203723

21. Liu Y, Liu H, Bian Q, Guan Y. A 67-Year-Old Man with Grade 3 Reactive Cutaneous Capillary Endothelial Proliferation Induced by Camrelizumab First Manifested in the Oral Mucosa - A Case Report. Int J Surg Pathol. 2024;32(4):803-9. http://doi.org/10.1177/10668969231195032

22. Hui HZ, Wang YJ, Cheng JR, Mao H, Jiang X, Guo HX, et al. Camrelizumab-Associated Psoriasis. Am J Ther. 2023;30(5):e461-3. http://doi.org/10.1097/MJT.0000000000001591

23. Kang Y, Zhen H, Ma N, Zhao H, Cao B. Encephalitis in a patient with hypopharynx cancer treated with immune checkpoint inhibitors and radiotherapy: a case report and review of the literature. J Cancer Res Clin. 2023;149(17):16239-46. http://doi.org/10.1007/s00432-023-05328-3

24. Li F, Wang T, Tang F, Liang J. Fatal acute-on-chronic liver failure following camrelizumab for hepatocellular carcinoma with HBsAg seroclearance: a case report and literature review. Front Med-Lausanne. 2023;10:1231597. http://doi.org/10.3389/fmed.2023.1231597

25. Jin Y, Xu J, Zhuang D, Dong L, Sun Y, Zhao L, et al. Hepatic cavernous hemangioma developed in non-small cell lung cancer patients after receiving Camrelizumab treatment: two case reports. Front Oncol. 2023;13:1221309. http://doi.org/10.3389/fonc.2023.1221309

26. Xie XH, Shen PX, Wu JH, Qiu GH, Lin XQ, Xie ZH, et al. Recurrent pleural effusion as a rare manifestation after prolonged PD1 inhibitor (camrelizumab)-based immunotherapy: A case report. Hum Vacc Immunother. 2023;19(2):2240689. http://doi.org/10.1080/21645515.2023.2240689

27. Liu J, Cao G, Zhang G, Liu S, Shi D. Nasal alar metastasis of advanced hepatocellular carcinoma misdiagnosed as reactive cutaneous capillary endothelial proliferation in a patient treated with camrelizumab and apatinib: a case report. J Gastrointest Oncol. 2023;14(3):1643-9. http://doi.org/10.21037/jgo-23-336

28. Fu S, Li C, Wang Z, Zhong Z, Zhong Y. A case report of thalidomide in the treatment of camrelizumab-induced reactive cutaneous capillary hyperplasia. Medicine. 2023;102(26):e34120. http://doi.org/10.1097/MD.0000000000034120

29. Cheng S, Yang Y, Yu J, Chen W, Li X. Immune-Related Colitis Induced by Camrelizumab: A Case Report. J Inflamm Res. 2023;16:1727-31. http://doi.org/10.2147/JIR.S405023

30. Wang C, Lei K, Jia Y, Jiang Z, Wang S. Complete remission of reactive cutaneous capillary endothelial proliferation caused by the programmed cell death-1 inhibitor camrelizumab achieved through thalidomide monotherapy: A case report. Exp Ther Med. 2023;26(1):324. http://doi.org/10.3892/etm.2023.12023

31. Hua Y, Huang X, Li C, Gao N. An epulis-like camrelizumab related reactive cutaneous capillary endothelial proliferation (RCCEP) in the oral cavity: A case report. Oral Oncol. 2023;140:106369. http://doi.org/10.1016/j.oraloncology.2023.106369

32. Zhang Y, Cui Y, Li Y, Cong L. Immune Checkpoint Inhibitor-Induced Primary Hyperparathyroidism in a Small-Cell Lung Cancer Patient: A Case Report. Medicina-Lithuania. 2023;59(2). http://doi.org/10.3390/medicina59020215

33. Wei T, Wang Z, Liu X. Adverse reactions and efficacy of camrelizumab in patients with lung adenocarcinoma with high PD-L1 expression: A case report. Medicine. 2023;102(7):e32731. http://doi.org/10.1097/MD.0000000000032731

34. Mei H, Wen W, Fang K, Xiong Y, Liu W, Wang J, et al. Immune checkpoint inhibitor-induced myocarditis and myositis in liver cancer patients: A case report and literature review. Front Oncol. 2022;12:1088659. http://doi.org/10.3389/fonc.2022.1088659

35. Rao H, Guo Z, Wen X, Zeng X, Wu L, Huang L. Case Report: Immune checkpoint inhibitor-related vitiligo-like depigmentation in non-melanoma advanced cancer: A report of three cases and a pooled analysis of individual patient data. Front Oncol. 2022;12:1099108. http://doi.org/10.3389/fonc.2022.1099108

36. Gao L, Li X, Guo Z, Tang L, Peng J, Liu B. Immune checkpoint inhibitor-induced myocarditis with myasthenia gravis overlap syndrome: A case report and literature review. Medicine. 2022;101(49):e32240. http://doi.org/10.1097/MD.0000000000032240

37. Li J, Yi S, Huang Y, Peng Y, Yang Z. Anti-TIF1-gamma antibody-positive dermatomyositis caused by camrelizumab in a patient with oesophageal cancer. Clin Exp Rheumatol. 2023;41(2):395. http://doi.org/10.55563/clinexprheumatol/knb47b

38. Zhan Y, Zhao W, Ni K, Liu Z, Su Y, Li X, et al. Case report: Camrelizumab associated with central retinal vein occlusion. Front Immunol. 2022;13:1025125. http://doi.org/10.3389/fimmu.2022.1025125

39. Shen X, Yang M, Xu H, Zhou H, Wang L, Ma J. Immunotherapy-Associated Hypophysitis under Anti-PD1: Two Case Reports. Endocr Metab Immune. 2023;23(7):996-1004. http://doi.org/10.2174/1871530323666221208111823

40. Wu C, Liu W, Pu J, Feng T, Chang Y, Wang X, et al. Fractional exhaled nitric oxide in checkpoint inhibitor pneumonitis: a case report and literature review. Immunotherapy-Uk. 2022;14(17):1361-7. http://doi.org/10.2217/imt-2022-0094

41. Zhang X, Shu X, Long B. Leptomeningeal metastasis from de novo metastatic nasopharyngeal carcinoma: a case report. Transl Cancer Res. 2022;11(9):3349-56. http://doi.org/10.21037/tcr-22-1211

42. Han X, Meng M, Zhang T, Wang J, Huang G, Ni Y, et al. Hypophysitis: A rare but noteworthy immune-related adverse event secondary to camrelizumab therapy. J Cancer Res Ther. 2022;18(5):1440-3. http://doi.org/10.4103/jcrt.jcrt_831_21

43. Wu R, Ju Y, Long T, Su Z, Zhu G, Liu S. Anlotinib improved the reactive cutaneous capillary endothelial proliferation induced by camrelizumab: a case report. Transl Cancer Res. 2022;11(8):2940-5. http://doi.org/10.21037/tcr-22-426

44. Hou Y, Su Q, Tang S, Li H. Camrelizumab-Induced Isolate Abducens Neuritis: A Rare Ophthalmic Immune-Related Adverse Events. Brain Sci. 2022;12(9). http://doi.org/10.3390/brainsci12091242

45. Wu PS, Xiong D, Feng YB, Xiang L, Zhu J. Case report: A case of acute mastitis associated with reactive cutaneous capillary endothelial proliferation after camrelizumab treatment: A new immune-related adverse event. Front Immunol. 2022;13:939873. http://doi.org/10.3389/fimmu.2022.939873

46. Gao Z, Xu Y, Zu J, Wang X, Sun C, Qiu S, et al. The time window for the reversal of depigmentation from aggravation to recovery in a non-small-cell lung cancer patient with pre-existing vitiligo using anti-programmed cell death-1 therapy: A case report. Front Immunol. 2022;13:946829. http://doi.org/10.3389/fimmu.2022.946829

47. Zhang B, Gyawali L, Liu Z, Du H, Yin Y. Camrelizumab-Related Lethal Arrhythmias and Myasthenic Crisis in a Patient with Metastatic Thymoma. Case Rep Cardiol. 2022;2022:4042909. http://doi.org/10.1155/2022/4042909

48. Lin C, Li X, Qiu Y, Chen Z, Liu J. PD-1 inhibitor-associated type 1 diabetes: A case report and systematic review. Front Public Health. 2022;10:885001. http://doi.org/10.3389/fpubh.2022.885001

49. Liu K, Bao JF, Wang T, Yang H, Xu BP. Camrelizumab-induced anaphylactic shock in an esophageal squamous cell carcinoma patient: A case report and review of literature. World J Clin Cases. 2022;10(18):6198-204. http://doi.org/10.12998/wjcc.v10.i18.6198

50. Guo K, Chen M, Li J. PD-1 Inhibitor-Induced Thyrotoxicosis Associated with Coronary Artery Spasm and Ventricular Tachycardia. Cardiovasc Toxicol. 2022;22(10-11):892-7. http://doi.org/10.1007/s12012-022-09756-4

51. Ye D, Qiu M, Wu L, Li B. Clinical Features of Camrelizumab-Associated Diabetes Mellitus. Am J Ther. 2023;30(6):e559-61. http://doi.org/10.1097/MJT.0000000000001493

52. Yang Y, Li J, Till BG, Wang J, Zhang B, Wang H, et al. Toxic Epidermal Necrolysis-Like Reaction Following Combination Therapy With Camrelizumab and Apatinib for Advanced Gallbladder Carcinoma. Front Oncol. 2021;11:728253. http://doi.org/10.3389/fonc.2021.728253

53. Bai J, Li D, Yang P, Xu K, Wang Y, Li Q, et al. Camrelizumab-Related Myocarditis and Myositis With Myasthenia Gravis: A Case Report and Literature Review. Front Oncol. 2021;11:778185. http://doi.org/10.3389/fonc.2021.778185

54. Zhou J, Mao Q, Li Y, Li Z, He H, Chen Q, et al. Oral reactive capillary hemangiomas induced by SHR-1210 in the treatment of non-small cell lung cancer: a case report and literature review. Bmc Oral Health. 2021;21(1):559. http://doi.org/10.1186/s12903-021-01901-9

55. Zhao LZ, Liu G, Li QF, Chen G, Jin GW. A case of carrelizumab-associated immune myocarditis. Asian J Surg. 2022;45(1):496-7. http://doi.org/10.1016/j.asjsur.2021.08.067

56. Chen Y, Jia Y, Liu Q, Shen Y, Zhu H, Dong X, et al. Myocarditis related to immune checkpoint inhibitors treatment: two case reports and literature review. Ann Palliat Med. 2021;10(7):8512-7. http://doi.org/10.21037/apm-20-2620

57. Zhang C, Qin S, Zuo Z. Immune-related myocarditis in two patients receiving camrelizumab therapy and document analysis. J Oncol Pharm Pract. 2022;28(6):1350-6. http://doi.org/10.1177/10781552211027339

58. Tan YW, Chen L, Zhou XB. Efficacy of artificial liver support system in severe immune-associated hepatitis caused by camrelizumab: A case report and review of the literature. World J Clin Cases. 2021;9(17):4415-22. http://doi.org/10.12998/wjcc.v9.i17.4415

59. Li L, Lou A, Yu J. Immune checkpoint inhibitor-related pneumonitis induced by camrelizumab: a case report and review of literature. Ann Palliat Med. 2021;10(7):8460-6. http://doi.org/10.21037/apm-21-23

60. Tan Y, Ye Y, Chen L. Fatal immune-related hepatitis with intrahepatic cholestasis and pneumonia associated with camrelizumab: A case report and literature review. Open Med-Warsaw. 2021;16(1):553-7. http://doi.org/10.1515/med-2021-0267

61. Wei Z, Yang X, Ye X. Rechallenge of camrelizumab in non-small-cell lung cancer patients treated previously with camrelizumab and microwave ablation. J Cancer Res Ther. 2020;16(5):1191-5. http://doi.org/10.4103/jcrt.JCRT_798_20

62. Wang D, Zhang S, Ding P, Zhao Y, Zhang X, Zhao Q. Immune-Related Adverse Events Mimicking Behcet's Disease in a Gastric Cancer Patient Following Camrelizumab Treatment. Iran J Immunol. 2020;17(2):167-71. http://doi.org/10.22034/iji.2020.85507.1717

63. Chen Y, Huang Z, Xing L, Meng X, Yu J. Radiation Recall Pneumonitis Induced by Anti-PD-1 Blockade: A Case Report and Review of the Literature. Front Oncol. 2020;10:561. http://doi.org/10.3389/fonc.2020.00561

64. Wang F, Sun X, Qin S, Hua H, Liu X, Yang L, et al. A retrospective study of immune checkpoint inhibitor-associated myocarditis in a single center in China. Chin Clin Oncol. 2020;9(2):16. http://doi.org/10.21037/cco.2020.03.08

65. Yu Q, Wang WX. Camrelizumab (SHR-1210) leading to reactive capillary hemangioma in the gingiva: A case report. World J Clin Cases. 2020;8(3):624-9. http://doi.org/10.12998/wjcc.v8.i3.624

66. Xu Y, Cai Y, Zu J, Wang X, Wang Y, Sun C, et al. Aggravation of depigmentation for a non-small-cell lung cancer patient with pre-existing vitiligo using anti-programmed cell death-1 therapy: case report. Immunotherapy-Uk. 2020;12(3):175-81. http://doi.org/10.2217/imt-2019-0090

67. Teng Y, Guo R, Sun J, Jiang Y, Liu Y. Reactive capillary hemangiomas induced by camrelizumab (SHR-1210), an anti-PD-1 agent. Acta Oncol. 2019;58(3):388-9. http://doi.org/10.1080/0284186X.2019.1567935
