# Supplementary material for: Safety profile of camrelizumab: An analysis based on literature and database review
Source: PLoS One. 2026 Jul 23;21(7):e0354252. doi: 10.1371/journal.pone.0354252 (PMC13395421; doi:10.1371/journal.pone.0354252)
Supplement: S2 Supplementary — (DOC) [file pone.0354252.s002.doc]

| **ID** | **Clinical symptoms** | **Laboratory testing** | **CT and MRI** | **Treatment** | **Outcome** |
| --- | --- | --- | --- | --- | --- |
| 1 | Pruritus (significant itching), erythematous rashes (on face, neck, trunk, and upper limbs), recurrent oral ulcers, xerosis, hypopigmentation, and scaly skin | Anti-SSA/Ro60KD antibody weakly positive,ANA positive (1:320, granular pattern),anti-dsDNA antibody negative (18.582 IU/ml),anti-SSA/Ro52KD, anti-SSB/La, anti-SmD1, and histone antibodies negative,renal function and muscle enzymes normal,Skin biopsy (right upper arm): Hyperkeratosis, increased melanin granules in basal layer, scattered liquefactive degeneration, perivascular and periappendageal lymphocyte-predominant inflammatory cell infiltration in dermis | Chest CT scans showed central lung cancer in the right lower lobe with obstructive pneumonia, segmental atelectasis, mucus plugs, and hilar lymph node enlargement | Discontinuation of Camrelizumab,local corticosteroids,systemic glucocorticoids (prednisone 20 mg daily for 2-4 weeks, tapered to 5 mg maintenance dose for total 6 months),hydroxychloroquine (0.2 g twice daily),photoprotection measures,switched to radical radiotherapy for primary cancer | Pruritus significantly relieved after 5 days of treatment,rash decreased after 2 weeks;overall symptom resolution |
| 2 | Dyspnea,Dizziness, chest tightness, pruritic erythematous macules (especially on upper limbs), hypotension | BP: 76/42 mmHg, HR: 83 bpm, RR: 15/min, SpO2: 94%. After treatment, BP increased to 110/80 mmHg | Baseline PET/CT showed bone destruction at clivus. Follow-up nasopharyngeal MRI after treatment showed reduction in skull base lesion | Immediate discontinuation of camrelizumab infusion. Intravenous dexamethasone (10 mg), intramuscular diphenhydramine, calcium gluconate, and 500 mL normal saline.Successful rechallenge with slow infusion of camrelizumab (started at 10 drops/min, increased to 30 drops/min after 30 min).Subsequent immunotherapy changed to tislelizumab (0.2 g) with prophylactic antihistamines (promethazine, dexamethasone, loratadine) | Successful rechallenge without adverse reactions |
| 3 | Pruritic erythematous rash on hands, feet, scrotum,Painful blisters (0.5x0.5 cm to 3x3 cm), >30% body surface area (BSA) involvement,Swelling in affected areas | Squamous cell carcinoma antigen: ↑5.07 ng/mL (normal not specified),CRP, liver/kidney function, electrolytes, coagulation, cardiac markers, thyroid function: Normal,Drainage fluid culture: Negative | Chest MRI: Confirmed esophageal carcinoma progression (cT4bNxM1) | Glucocorticoids: IV methylprednisolone (80mg→20mg tapered over 7 days) → IV dexamethasone (18mg→11.25mg tapered),Topical therapy: Unspecified blister/skin care,Supportive: Gastric mucosal protection (hydrotalcite chewable tablets), nutrition support,Oral methylprednisolone post-discharge,Permanent discontinuation of camrelizumab | Full skin recovery |
| 4 | Fever,Generalized body pain,Diffuse erythematous rash,Blisters/ulcerations,Oral mucosal ulcers,Nikolsky sign (+) | Immunophenotyping: ↓ T cells/CTLs/NK cells,↑ IL-10 ,Histopathology: Epidermal detachment, inflammatory infiltrate | CT: LUAD progression (SD) | High-dose methylprednisolone (80mg → 500mg taper), IVIG (22.5g × 8 days),Epidermal growth factor, clobetasol | Skin: Rash resolved with desquamation (no recurrence) |
| 5 | Fatigue,Refractory hypokalemia (CTCAE grade 3) | Persistent hypokalemia (2.58-2.85 mmol/L),Hyperchloremia (111-115.1 mmol/L),Metabolic acidosis (pH 7.34,HCO:12.2 mmol/L),ANA+ (1:100),Normal renal/thyroid/cortisol function | Not performed for acute presentation | Potassium citrate supplementation,Hydroxychloroquine (200mg bid),Permanent camrelizumab discontinuation after rechallenge failure | Symptom recurrence upon camrelizumab rechallenge,Sustained remission after permanent discontinuation |
| 6 | Asymptomatic troponin elevation | Cardiac Biomarkers:Hs-TnT: 653 pg/mL,CK-MB: 22.6 ng/mL,Mb: 142 ng/mL,NT-proBNP: 798 pg/mL | ECG: Normal - Echo: Normal | Methylprednisolone 500mg IV daily ×4d,Reduced to 240mg→40mg, then oral prednisone | Biomarkers normalized,No recurrence at follow-up |
| 7 | Diarrhea,Fever | Cardiac Biomarkers:Hs-TnT: 575 pg/mL,CK-MB: 33.9 ng/mL,NT-proBNP: 15,497 pg/mL | ECG: RBBB + LAHB, ST-T abnormalities,Echo: Segmental dyskinesia, ventricular thickening | Methylprednisolone 500mg IV daily ×5d,IVIG 10g/day (added Day 5),Blood transfusion, hemostasis | Died (Day 8) due to myocarditis + heart failure + GI bleed |
| 8 | Dyspnea,Profuse sweating, hypoxia | Hs-TnT: 174 pg/mL,NT-proBNP: 10,117 pg/mL,PCT: 6.81 ng/mL,IL-6: 43.1 pg/mL | ECG: Borderline QT prolongation, ST-T changes,Echo: LV anterior/posterior wall thinning, akinesia (LVEF 54%) | Methylprednisolone 240mg IV daily ×7d,IVIG 20g/day ×3d (added Day 7),Reduced to 160mg→oral prednisone | Biomarkers improved after IVIG,Discharged on tapering steroids |
| 9 | Severe systemic fatigue,Limb numbness,Walking instability | CSF analysis: Total protein ↑1,399 mg/L (normal: 50–150 mg/L),Glucose: 4.7 mmol/L (normal: 6.0 mmol/L) | T2-FLAIR: Patchy high-signal shadows in bilateral frontal/parietal lobes | Neurotrophic drugs (thioctic acid + cobamamide,ineffective),Methylprednisolone IV: 500mg/day (days 1–4) → 120mg/day (days 5–10) → 60mg/day (days 11–15),Oral prednisone acetate: 30mg/day (days 16–30) | Symptoms resolved completely,No recurrence of neurological symptoms at 1-year follow-up |
| 10 | Diarrhea:Severe non-bloody (7–8 episodes/day),Fecal occult blood positive | Fecal calprotectin ↑61.4 μg/g (normal <50 μg/g),CRP ↑17 mg/L (normal <8 mg/L) | CT: Complete HCC necrosis, perihepatic effusion, transverse colon mucosal edema,Colonoscopy: Diffuse congestion/edema/ulcers (descending/transverse colon),Histology: Active colitis with inflammatory infiltrate | Methylprednisolone IV (80mg→40mg→24mg→tapered withdrawal → recurrence),Vedolizumab 300mg IV ×2 doses (2-week interval) | Resolved after vedolizumab,No recurrence at 2.5 years |
| 11 | Fever(low-grade in 1st episode, high-grade in 2nd),Abdominal pain:Periumbilical (1st), upper abdominal dull pain (2nd)Tenderness: Periumbilical (1st), right upper quadrant (2nd) | Lab testing (1st episode):WBC↑ (25.10×10?/L), NEUT↑ (22.21×10?/L), CRP↑ (150.66 mg/L), PCT↑ (0.91 ng/mL),normal LFTs,(2nd episode):WBC↑ (11.97×10?/L), NEUT↑ (10.78×10?/L), CRP↑ (150.66 mg/L), PCT↑ (2.79 ng/mL),LFTs↑ (ALT 775 U/L, AST 551 U/L, ALP 235 U/L, GGT 225 U/L, TBil 69.03 μmol/L, DBil 50.03 μmol/L) | CT/MRI (1st):Gallbladder enlargement, thickened wall ,(CT)MRI (2nd):Gallbladder enlargement, wall thickening (1 cm), common bile duct narrowing,mild bile duct dilation | 1st & 2nd cholecystitis:Conservative (antibiotics, acid inhibitors, antispasmodics),resolved in 1 week,Camrelizumab discontinued after 8th cycle,S-1:2 cycles (discontinued due to toxicity),Immunotherapy rechallenge:Zimberelimab (240 mg q2w) for 37 cycles | Cholecystitis resolved with conservative treatment,Tumor remained in complete remission (PFS 28.8 months) |
| 12 | Severe progressive fatigue,Progressive dysphagia | Platelet count <25×10^9^/L,NGS: TP53 mutation(p.L114Afs*34, 57.93% abundance), JAK2/KRAS copy number gain (>20 copies),IHC/IF: CD68+ macrophage infiltration, JAK2 overexpression | Liver/cervical lymph node metastases→Diffuse liver lesions, multiple lung metastases | Corticosteroids (1 mg/kg/day) → ineffective | Thrombocytopenia refractory to all interventions,Rapid disease progression with new lung/liver metastases,Death from multi-organ failure |
| 13 | Unconsciousness (10 min post-reaction),Severe weakness,Sweating, pale complexion,Clammy/cyanotic limbs, Generalized pruritic erythema (especially upper limbs) | ↑ Eosinophils (36.8%),↑ D-dimer (0.61 mg/L),↑ hs-CRP (4.4 mg/L),Normal liver/kidney function,SpO₂: 75% → 82%,BP: 86/58 mmHg → 79/49 mmHg,HR: 86 bpm → 83 bpm | NA | Oxygen supplementation,Methylprednisolone (80mg IV) + Dopamine infusion,Dexamethasone (5mg IV, initial) | Consciousness regained within 20 min:BP stabilized (122/84 mmHg) within 2h,SpO₂ normalized (100%),Skin erythema resolved completely |
| 14 | Lethargy (during adrenal crisis),Weight loss (5kg/week),Nausea/vomiting,Polyuria/polydipsia | TSH >100 μIU/mL, TPOAb↑, TgAb↑,Adrenal: Cortisol↓ (3.41 μg/dL), ACTH↑ (313.16 pg/mL),Fasting glucose↑ (25.72 mmol/L), C-peptide↓ (0.15 ng/mL), K?↑ (5.66 mmol/L), Na↓ (126 mmol/L),Autoantibodies: All diabetes-related antibodies negative | Thyroid/Adrenal ultrasound: Normal | DKA: IV fluids + insulin,Adrenal crisis: Hydrocortisone IV (200mg/day),Maintenance:Levothyroxine (75μg/day),Hydrocortisone (10mg AM + 5mg PM),Insulin glargine (7U) + aspart (4U TID) | Symptoms resolved with hormone replacement,HbA1c: 7.6% at 6 months,Sustained hormone dependence |
| 15 | Asymptomatic (incidental finding on imaging) | Pathology: Biopsy-confirmed cavernous hemangioma | Dynamic enhancement showing progressive contrast filling (cavernous hemangioma) | Camrelizumab discontinued after biopsy complication (massive bleeding),Hemostasis + transfusion | Hemangioma size reduced after camrelizumab discontinuation |
| 16 | Asymptomatic,concurrent anal/oral RCCEP | Pathology:Anal capillary hemangioma (surgically excised) | Three enhanced lesions (gastric fundus varices + hepatic hemangiomas) | Apatinib reduced skin RCCEP but not visceral lesions | Hemangioma size reduced after 5.5 months off camrelizumab,Died of COVID-19 complications |
| 17 | Asymptomatic | Pathology:Moderately to poorly differentiated adenocarcinoma | Progressive enhancement pattern Confirmed hepatic hemangioma | Camrelizumab continued until progression,Lesions resolved spontaneously | Hepatic lesions resolved by 16-month follow-up |
| 18 | Asymptomatic | Immunohistochemical (IHC) staining results were positive for HER2 (2+) and PD-L1 (CPS 5) | Atypical arterial-phase enhancement in liver | Camrelizumab discontinued | Unknown |
| 19 | Asymptomatic | Pathology:IVb cervical cancer, classified as adenocarcinoma | Hepatic hemangioma | Camrelizumab discontinued | Unknown |
| 20 | Asymptomatic | Endoscopy: cauliflower-like tumor,Pathology:High to moderately differentiated squamous cell carcinoma | Atypical venous-phase enhancement in liver | Camrelizumab discontinued | Unknown |
| 21 | Diarrhea (Grade II),Rash/pruritus (progressive),Oral ulcer (Grade II) | FISH amplification (HER2/CEP17=5.8) | Liver metastases + nodal recurrence | Tislelizumab (PD-1) + trastuzumab (4 cycles, PR → PD),Disitamab vedotin (HER2-ADC) + tislelizumab (ongoing, SD) | Improved |
| 22 | Erosions of oral mucosa/lips,Hemorrhagic crusting,Erythema with purpuric centers on hands/feet | NA | NA | Discontinuation of camrelizumab + prednisolone | Improved |
| 23 | Extensive epidermal detachment (face/trunk/proximal limbs),Atypical target lesions (distal limbs),Nikolsky's sign positive,Oral/vulval mucosal erosions | NA | NA | ICU care: Methylprednisolone (120 mg/day) + IV immunoglobulin | Died (cardiac failure) |
| 24 | Papillary red lesions on eyelids/forehead/scalp,Eye irritation/foreign body sensation | PD-L1 CPS: 35,Histology: Proliferating fusiform/ovoid cells forming spaces | OCT angiography: Normal | Surgical excision of eyelid lesions | Resolved |
| 25 | Peanut-sized nodule on right lower eyelid causing incomplete eye closure,Scattered red spots on head/face/trunk | Histology: Nodular/lobulated capillary proliferation with RBC-filled lumens | Head CT: Nodule protruding outward | Surgical excision | Resolved |
| 26 | Diarrhea (20-30/day),bloody stools,Bilateral knee pain,Abdominal pain | ↑ CRP (89.1 mg/L), ↓ albumin (26 g/L),Stool: RBC/WBC 3+/HP,Colonoscopy: Pan-colonic inflammation, mucosal bleeding | MRI: Knee inflammatory exudation | Camrelizumab discontinued,Mesalazine (oral) + dexamethasone enema,Anti-inflammatory plasters for knees | Symptoms improved (diarrhea reduced, no bloody stool) |
| 27 | Fatigue,reactive cutaneous capillary endothelial proliferation | Testing: ECG ST elevation, NT-proBNP ↑, cTnT ↑ | Echocardiography, cardiac MRI, angiography | Immunoglobulin, methylprednisolone, compound danshen,camrelizumab withheld | Topical glucocorticoid,immunotherapy continued |
| 28 | Pain impacting eating,speaking, sleeping | Testing: Histopathology, flow cytometry CD4+ ↑ | NA | Full recovery, asymptomatic at 1 year | Improvement after 2 weeks, stable condition |
| 29 | Bleeding lesions upon touch,tingling sensation after rupture,psychological burden (fear of suffocation from oral lesions) | Histological examination: Large number of proliferating capillaries, capillary endothelial cell proliferation, nutrient vessels with larger lumen. Hematoxylin and eosin (H&E) staining confirmed diagnosis. | NA | Discontinuation of Camrelizumab,excision of two nodules under local anesthesia,compression hemostasis for ruptured lesions,anti-infection with topical fusidic acid cream (twice daily),chlorhexidine gargle for oral cleaning (three times daily). | Gradual improvement at 4 weeks after discontinuation,mostly relieved at 7 weeks after discontinuation,RCCEP remission observed. |
| 30 | Psoriasis-like scaly erythema and plaques on upper extremities and scalp,positive Auspitz sign. | Three-dimensional skin CT: Revealed Munro's microabscess, psoriasis-like hyperplasia, squamous epithelium, and dermal papilla vascular dilation. | NA | Topical glucocorticoid treatment | Lesions improved after 2 weeks of topical glucocorticoid treatment. |
| 31 | Generalized fatigue and weakness,Visual hallucinations,Tremors,loss of appetite | Hyponatremia (128 mmol/L),CSF: ↑ Protein (59.64 mg/dL), ↑ IgG (49.50 mg/L), ↑ IgG index (1.00), ↑ IgG-syn (13.63 mg/24h),Blood: Hyponatremia (128 mmol/L), | Brain MRI: T2-FLAIR hyperintense signal in right temporal lobe,Radiotherapy dose: Bilateral temporal lobes (2.11 Gy), whole brain (25.28 Gy) | IV methylprednisolone (120 mg/day × 3 days → taper over 6 weeks),Olanzapine (2.5 mg/night) for hallucinations,Permanent cessation of Camrelizumab | Symptoms improved |
| 32 | severe weakness,Encephalopathy | Dynamic changes in liver/kidney function:↑AST/ALT (300/722 U/mL),↑TBil/DBil (21.84/16.88 mg/dL),↑PT/INR (20.9 s/1.84),↑Cr (1.05 → 3.54 mg/dL) | No biliary obstruction/intrahepatic progression | Camrelizumab discontinuation,Corticosteroids (methylprednisolone),Plasma dialysis (4 sessions),Tenofovir alafenamide (TAF),Antibiotics (ertapenem) | Death due to ACLF, renal failure, septic shock |
| 33 | Right upper quadrant discomfort | ↑CEA (71.5 ng/mL), ↑CA24-2 (89 U/mL),Post-biopsy hemorrhage | Hepatic nodules (cavernous hemangioma confirmed by biopsy) | Camrelizumab discontinuation Switch to Tislelizumab | Hemangioma resolved |
| 34 | NA | NA | Hepatic nodules (cavernous hemangioma confirmed by biopsy) | Camrelizumab discontinuation | Hemangioma resolved |
| 35 | Pleural and pericardial effusion | Cytology analysis (negative for tumor cells), IHC staining (TTF-1+, Napsin-A+, CK7+), NGS analysis (KRAS, TP53 mutations) | Chest CT showing PE, pericardial effusion, filling defects | Camrelizumab + chemo continued,intrathoracic dexamethasone injections | PE reduced after dexamethasone,SD maintained |
| 36 | RCCEP (head, neck, trunk, limbs),Nasal alar lesion: Mulberry-like→tumor-like, recurrent bleeding | AFP: 5.49 ng/mL (baseline) | Abdominal CT/MRI: Multiple active intrahepatic lesions,Retroperitoneal lymph node metastases,Chest CT:Pulmonary metastasis | Surgical resection (Jan 2021) → Radiotherapy (6,000 cGy/30f),Continued camrelizumab + apatinib + TACE/ablation | Symptoms improved |
| 37 | Psychological distress,Scattered red/red-black papules (face, neck, back),Ulcerated eyelid lesion | NA | NA | Oral thalidomide (50 mg AM + 100 mg PM) | RCCEP resolved after 3 weeks |
| 38 | 5–6 episodes of thin mucus/bloody stools,Hematochezia | Stool OB+, RBC 2/HP,WBC: 2.93×10⁹/L,CRP: 4.42 mg/L Multiple flake congestion/edema in terminal ileum/colon,chronic inflammation | Abdominal CT: No morphologic abnormalities | Loperamide (symptomatic) Sulfasalazine (0.25g bid ×6 weeks) Interrupted, resumed later | Diarrhea/bloody stool resolved,partial tumor control |
| 39 | Facial/torso vascular nodules (1–5mm), bleeding on contact | NA | Peritoneal/vaginal metastases | Thalidomide (100mg nightly ×1 month),Continued without interruption | Complete resolution of RCCEP |
| 40 | Painless nodule on gingiva, skin lesions | Histopathological examination: Epithelial erosion, capillaries proliferation,No bone destruction on radiographic exam | Radiographic exam: No specific bone destruction | Surgery for oral lesion and one skin lesion,ligation for other lesions | Good prognosis,no recurrence during follow-up |
| 41 | Dysphagia, inappetence, urination difficulties | Elevated ACTH (109.7 pg/mL), cortisol (999.4 nmol/L), ferritin (1111.6 ng/mL), hypercalcemia (3.01 mmol/L), hypophosphatemia (0.47 mmol/L), elevated PTH (300.5 pg/mL) | Lumbar enhanced CT and bone scan: No metastasis | Methylprednisolone (80 mg daily) | Died of respiratory failure due to progressive malignancy and secondary infection |
| 42 | Yellow staining of sclera and skin, itching throughout body, dry mouth, ecchymosis of skin (right lower limb), brown urine, pale stools ("white potting soil"), poor appetite | Abnormal liver function: Total bilirubin (TB) 245.9 μmol/L, direct bilirubin (DB) 227.7 μmol/L, alanine aminotransferase (ALT) 56.9 U/L, aspartate aminotransferase (AST) 97 U/L, alkaline phosphatase (ALP) 815 U/L,decreased adrenocorticotropic hormone (ACTH) 0.13 pg/mL and triiodothyronine (T3) 1.03 nmol/L,increased brain natriuretic peptide (BNP) 259.9 pg/mL | Chest CT: Tumor size changes (11.0x8.7 cm at diagnosis to 8.7x4.5 cm at day 59 post-treatment),MRI: Confirmed brain metastases | Methylprednisolone (initial 2 mg/kg, escalated to 8 mg/kg), mycophenolate mofetil (1g twice daily), plasma exchange (3 days), ceftriaxone (for infection prevention), esomeprazole (gastric protection), vitamin D and calcium supplementation,camrelizumab discontinued | Immune-related adverse events (irAEs) controlled,tumor showed partial response (PR) with continued shrinkage after nearly 2 months without treatment,patient stabilized |
| 43 | Dyspnea,Chest tightness, expectoration (whitish sputum), decreased muscle strength in both lower extremities | Elevated creatine kinase (CK) 1572.1 U/L, creatine kinase isoenzyme (CK-MB) 193.6 U/L, lactate dehydrogenase (LDH) 744.0 U/L, myoglobin >900 μg/L,alanine aminotransferase (ALT) 108.6 U/L, aspartate aminotransferase (AST) 146.3 U/L,normal cardiac troponin (cTnI) and brain natriuretic peptide (BNP) | Abdominal CT: Multiple nodular enhancements indicating tumor recurrence,MRI: Used for follow-up imaging | Methylprednisolone sodium succinate IV (steroid pulse therapy), gradually reduced doses,polyene phosphatidylcholine and omeprazole for supportive care,camrelizumab discontinued | Symptoms significantly improved,laboratory markers normalized,discharged on oral methylprednisolone,later recurrence treated with microwave ablation,overall fair condition with no new lesions at follow-up |
| 44 | Body itchiness | Wood's lamp examination (confirmed depigmentation)， biopsy (hyperkeratosis, dermal edema, decreased melanocytes),immunohistochemistry (S-100+, HMB45+, melan-A+) | Chest and abdominal CT (reexamined every two cycles for assessment),MRI (revealed multiple intracranial metastases) | Discontinued camrelizumab,high palliative radiotherapy,targeted therapy with apatinib,whole brain radiotherapy | Impaired consciousness,discharged,eventual death (implied from progression) |
| 45 | Limb weakness,Chest tightness | High-sensitivity cardiac troponin T (3015 pg/mL),NT-proBNP (5671 pg/mL),creatine kinase (1419 U/L),ECG (sinus tachycardia, premature beats),white blood cell and neutrophil counts elevated | Echocardiography (impaired left ventricular function),CT/MRI not specified | Intravenous methylprednisolone,oral prednisone,oxygen,isosorbide nitrate,sodium creatine phosphate,diuretics,antiarrhythmics,anticholinesterase drugs,immunoglobulin | Partial symptom relief,cardiac markers remained high,family refused treatment,died soon after |
| 46 | Swollen face,Erythema papules on cheeks, hands, bilateral hip, lower extremities,Scales and pruritus,Drinking face, heliotrope eruption,V-shape sign, shawl sign, Gottron's sign,Scaly erythematous eruption and depigmentation spots | Elevated fibrinogen, erythrocyte sedimentation rate (ESR), C-reactive protein (CRP),Positive antinuclear antibody (ANA), anti-TIF1-y antibody, anti-endothelial cell antibody,Histopathology: Perivascular infiltrate with lymphocytes, neutrophils, eosinophils | MRI of upper arm: Abnormally high signal intensity in muscles on T2 images,Chest enhanced CT: Earlier showed vertebral metastasis (not directly related to onset) | Camrelizumab discontinued,Methylprednisolone 40 mg/d ,IVIG 12.5 g + cyclophosphamide 0.8 g,Methylprednisolone + methotrexate 10 mg/week + oral prednisone | Cutaneous eruption and facial swelling improved,Sore limbs relieved,Symptoms resolved after treatment |
| 47 | Diminution of vision,Cystoid macular edema,Intraretinal hemorrhages,Vitreous opacities | Not specified for eye condition | Cranial CT: No intracranial metastasis,OCT: Cystoid exudation in macular area, fluid accumulation,B-scan ultrasonography:Vitreous opacities | Camrelizumab discontinued,Vitrectomy,phacoemulsification, intraocular lens implantation,Oral tanakan (ginkgo biloba extract) + compound anisodine injections | Vision limitedly restored,Symptoms in left eye improved after discontinuation |
| 48 | Severe anorexia,Adrenal nodules (metastatic adenocarcinoma) | Hormones: ↓ ACTH (<10 pg/mL), ↓ cortisol (AM: 14.08 nmol/L),24-h urine-free cortisol: 12.32 μg/24h | NA | Cortisone acetate (37.5 mg/day) | Anorexia improved,Pituitary lesion resolved,Died during sixth-line cancer therapy |
| 49 | Fatigue,Dyspnea,Productive cough, dry rales | PO_2_ 61 mm Hg, SaO_2_ 92%, normal leukocyte count, procalcitonin, BNP,FeNO 92 p.p.b | Chest CT showing reticular abnormalities and ground-glass opacities | Discontinued camrelizumab,budesonide, ipratropium bromide, acetylcysteine, doxofylline, methylprednisolone | Symptoms improved, FeNO decreased, partial remission of cancer |
| 50 | Transient unconsciousness,Persistent fatigue,Pain in both lower limbs | EBV-DNA ↑ (472,000 copies/mL → rebounded) | NA | Whole-brain radiotherapy (30 Gy/10 F) + Capecitabine | PFS: 5 months,intracranial lesions resolved |
| 51 | General malaise Appetite loss Hypophysitis (central adrenal insufficiency) | Cortisol (43.9 nmol/L), ↓ ACTH (<1.5 pg/mL), ↓ TSH (0.0466 µIU/mL) | NA | Methylprednisolone → Hydrocortisone maintenance | Symptoms resolved,ACTH/cortisol deficiency persisted |
| 52 | Fever,Syncope Hypophysitis (adrenal crisis) | ↓ Cortisol (0.134 nmol/L), ↓ ACTH (0.508 pg/mL), ↓ TSH (0.089 µIU/mL), Hyponatremia (122 mmol/L) | NA | Methylprednisolone → Prednisone maintenance | Symptoms resolved,ACTH/cortisol deficiency persisted |
| 53 | Multiple bright red "red-nevus-like" and "pearl-like" papules on face, head, neck, chest,one ruptured rash under left eye | PD-1: TPS=5%, CPS=6 | Chest CT showed lung lesion improvement | Oral anlotinib (8 mg/day) for 5 days | RCCEP resolved within 5 days,discharged |
| 54 | Isolated right abducens nerve palsy,Horizontal diplopia,incomplete right eye abduction | Serum tumor markers normal (CEA, CYFRA211, NSE, Pro-Grp),autoantibodies negative | MRI: Thickening and enhancement of right abducens nerve | Oral methylprednisolone (48 mg/day, tapered) without camrelizumab discontinuation | Diplopia resolved completely within 1 month |
| 55 | Breast pain/swelling,RCCEP ("mulberry-like" skin lesions) | Normal inflammatory markers | Breast ultrasound: Inflammatory changes ("finger-like" tubular formations) | Camrelizumab discontinuation | Mastitis resolved after discontinuation |
| 56 | Hypothyroidism-related,Hair depigmentation (scalp/eyebrows/eyelashes) | Hypothyroidism (CTCAE Grade 1) | Reflectance confocal microscopy: Loss of pigment rings Wood's light: Bright bluish-white depigmentation | Anti-PD1 discontinued after 2 years,Levothyroxine for hypothyroidism | Gradual repigmentation over 9+ months post-discontinuation |
| 57 | Fatigue,Dyspnea,Muscle weakness,Palpitation, poor appetite, respiratory muscle failure | Elevated troponin I (13.94 ng/mL), CK-MB (213.70 ng/mL), CK (8811.40 U/L), ALT (290 U/L), AST (805 U/L),elevated inflammatory markers (WBC 18.8 x10^9/L, CRP 15.03 mg/L),negative for pathogens and autoantibodies (Tables 1 and 2). ECG showed progressive conduction abnormalities (e.g., bifascicular block) | Chest CT revealed anterior mediastinal mass (44x51 mm) with pleural invasion and effusion. Cardiac MRI not performed due to uncontrolled heart rate and breathing | Intravenous methylprednisolone (started at 80 mg/day, increased to 1 g/day), IVIG (20 g/day), mechanical ventilation, temporary pacemaker insertion, antiarrhythmic drugs (amiodarone, lidocaine, β-blocker), antibiotics (piperacillin/tazobactam), and supportive care | Death on day 5 of hospitalization due to ventricular fibrillation and cardiac arrest. |
| 58 | Hyperosmolar hyperglycemic coma, polydipsia (inferred from sugary drink consumption) | Plasma glucose (1082 mg/dL), HbA1c (7.88%), fasting C-peptide (0.04 ng/mL), positive IA-2A,urinary glucose (3+), urinary ketone body (±),arterial blood gas: pH 7.44, HCO3 19.7 mmol/L | NA | Fluid replenishment, insulin therapy (insulin glargine initially, then insulin aspart and degludec), correction of electrolyte imbalances | Continued camrelizumab treatment after glycemic stabilization,dependent on insulin therapy with brittle diabetes |
| 59 | Anaphylactic shock,drowsiness,breathing difficulties,Generalized rash, palpitations, sense of dying | ECG monitoring (pulse rate, blood pressure, respiratory rate), blood tests (WBC, neutrophil count, hemoglobin, etc.) | Enhanced CT scan (showed chronic inflammation and pleural effusion) | Stopped camrelizumab,intravenous fluids, adrenaline, dexamethasone, calcium glucosate, noradrenaline | Symptoms completely relieved after 2 hours,patient refused further camrelizumab due to fear of recurrence |
| 60 | Thyrotoxicosis with coronary artery spasm and ventricular tachycardia,Palpitation | Thyroid function tests (elevated FT3, FT4,suppressed TSH), ECG, Holter monitoring (ST-segment elevation, ventricular tachycardia), lipid panel | Chest CT showed lung nodules | Stopped camrelizumab,antivasospasm drugs (isosorbide mononitrate, diltiazem), sedative (alprazolam), antiarrhythmic (mexiletine) | Symptoms relieved |
| 61 | NA | Fasting blood glucose was found to be as high as 12.79 mmol/L, HbA1c was slightly elevated to 6.69%, and urine glucose was found +++ and urine ketone + | NA | Switched from camrelizumab to chemotherapy | The fulminant ICPis-induced diabetes is irreversible |
| 62 | Fatigue,Intolerable pain in hands and feet with swelling,soreness in compression areas like hips, back, and armsPruritic maculopapular rash, blistering, Nikolsky's sign positive, loss of appetite, hip/back/arm rash worsening in load-bearing areas | SCORTEN score 4 (predicted mortality 58.3%),skin biopsy (epidermal hyperkeratosis, edema, apoptosis),immunohistochemistry (CD4+, CD8+ T-cell infiltration, no B/NK cells),CyTOF (reduced CD4+/CD8+ T-cells),cytokine levels (reduction in IL-2, IL-4, IL-6, IL-10, IL-17A, TNF-α,slight IFN-γ elevation) | PET-CT scan showed cancer progression | Methylprednisolone (80 mg daily initially), IVIG added (30 g daily for 5 days), methylprednisolone tapered over ~1 month | Rash resolved completely with epidermal exfoliation,cancer progressed,discharged and received fourth-line therapy (discontinued due to progression and neurotoxicity). Patient survived |
| 63 | Rapid breathing and dyspnea leading to type II respiratory failure,weakness and soreness in both lower extremities,Palpitations, tachycardia, ventilator-associated pneumonia (Klebsiella pneumoniae and Acinetobacter baumannii) | ECG (sinus tachycardia),cardiac enzymes (CK 3503.1 U/L, CK-MB 178.7 U/L, LDH 622.6 U/L, cTnI 0.35–0.89 ng/mL),liver function (ALT 154.3 U/L, AST 204.4 U/L),anti-AChR-Ab (10.94 nmol/L),blood gas analysis (respiratory acidosis),EMG (myogenic damage) | Cervical-thoracic CT showed esophageal carcinoma and lymph node metastasis,follow-up CT showed no change | Methylprednisolone (120 mg daily, tapered), pyridostigmine bromide (30 mg tid), IVIg, ventilator support for respiratory failure, antibiotics (cefoperazone/sulbactam + tigecycline) | Condition improved,muscle strength recovered,biomarkers normalized,discharged. |
| 64 | Skin: Purple, dome-shaped, bright red papules on face, chest, abdomen, and hand. Oral: Gingival hyperplasia, easy bleeding when touched, affecting eating and speaking | Proliferation of capillaries and vascular endothelial cells in a lobulated shape. Panoramic radiograph showed alveolar bone resorption from periodontitis | X-ray showed no specific bone destruction in gingival area,alveolar bone resorption from periodontitis | Periodontal treatment,followed by surgical resection of oral and skin lesions | Good prognosis |
| 65 | Fever,Fatigue,Dyspnea,Tachycardia, hypotension | Electrocardiogram (ECG): Atrial flutter. Cardiac ultrasound: Ejection fraction (EF) reduced to 45%, improving to 65% after treatment | Chest computed tomography (CT) showed no obvious infection focus | High-dose intravenous methylprednisolone therapy (500 mg on day 1, tapering doses over days 2–12) | Improved condition,EF normalized,discharged and medication withdrawn |
| 66 | Dyspnea,Palpitations, later developed fatal MG (eyelid ptosis, limb weakness, respiratory failure) | cTnT:0.806 ng/mL → 0.952 ng/mL,NT-proBNP:321 ng/L → 2,509 ng/L,CK-MB:81.8 ng/mL,Thyroid:↓Free T3/T4,Anti-RyR/Titin Ab:Positive | NA | Methylprednisolone 240 mg/d → 120 mg/d → 40 mg/d + IVIG | Respiratory failure → Died |
| 67 | Fatigue,Dyspnea,Abdominal distension/pain | cTnT:0.044 μg/L,NT-proBNP:818 ng/L → 15,692 ng/L,ECG:ST depression,T-wave inversion | NA | Methylprednisolone 160 mg BID + Diuretics | Ventricular fibrillation → Died |
| 68 | Dyspnea,Limb numbness/pain, blurred vision, eyelid drooping | cTnT:1.030 μg/L,CK-MB:>300.0 μg/L,Myoglobin:>3000 μg/L,NT-proBNP:575 ng/L,ECG:Atrial arrhythmias, ST depression | NA | Methylprednisolone 160 mg BID → Prednisone (tapered) | Symptoms resolved → Discharged |
| 69 | Acute liver failure Loss of appetite, yellow urine, jaundice, fatigue | Elevated ALT, AST, TBIL, ALP, GGT,PT 19s, INR 1.8,negative viral/mitochondrial antibodies | Abdominal ultrasound/MRCP ruled out biliary obstruction | Steroids (methylprednisolone 40 mg/day) ineffective,DPMAS + plasma exchange (PE):5 sessions,UDCA continued | Liver function normalized 4 weeks post-discharge,Capecitabine later resumed without recurrence |
| 70 | Asymptomatic | WBC:8.38×10⁹/L, neutrophils 84%, CRP 124.61 mg/L,negative cultures | Multiple bilateral ground-glass opacities | Oral prednisolone (40 mg/day) + antibiotics (fluconazole/moxifloxacin),Camrelizumab discontinued | Radiographic improvement |
| 71 | Fever,Dyspnea,Jaundice, yellow skin, dark urine | Abnormal liver function: TBIL 52.2 μmol/L, DBIL 38.8 μmol/L, ALT 176.7 U/L, AST 250.5 U/L, ALP 336 U/L, GGT 638 U/L,Liver biopsy: Immune injury with ductopenia,Prothrombin time 41s, activity 19%, INR 4.03,Negative for autoantibodies | Chest CT: Interstitial inflammatory lesions in both lower lungs,Abdominal CT: Heterogeneous enhancement of liver density | Methylprednisolone (adjusted to 40–80 mg/day),Mycophenolate mofetil (2 g/day),Ursodeoxycholic acid,Plasma exchange and double plasma molecular adsorption system (DPMAS),Antibiotics (cefoperazone sulbactam, caspofungin) | Death due to liver failure, cardiopulmonary failure, and septic shock |
| 72 | Asymptomatic radiographic findings | NA | NA | Methylprednisolone (2mg/kg + taper),Camrelizumab resumed →Grade 3 IRP recurrence after 3 cycles,Methylprednisolone reinitiated | IRP resolved with steroids |
| 73 | Cough and shortness of breath (during recurrence) | NA | NA | Methylprednisolone (2mg/kg + taper), Camrelizumab,Steroids | IRP resolved with steroids |
| 74 | Oral ulcers, penile ulcers, skin lesions on hands/feet resembling folliculitis/acne, abdominal incision erosion with purulent secretion | Elevated IL-8 (25.92 pg/mL) | NA | Discontinued camrelizumab,Prednisone acetate (40 mg/day),Thalidomide (50 mg/day),Continued systemic chemotherapy | Symptoms improved |
| 75 | Fever,Dyspnea,Cough, tigue | Blood/Sputum culture: No infection | CT: Patchy consolidation/GGO in irradiated area | Discontinued Camrelizumab,Prednisolone (80mg q12h, tapered) | Symptoms improved |
| 76 | NA | Biomarkers: ↑CK, ↑CK-MB, ↑MYO, ↑Troponin I,ECG/Echo: Normal | NA | Methylprednisolone (4mg/kg/d, tapered over 8 weeks) | Full recovery |
| 77 | Gingival overgrowth (maxilla/mandible),Magenta pedunculated mass (lingual mandible),Bleeding on palpation,Tooth mobility (Grade I-III) | Histopathology:Stratified squamous epithelium,vascular proliferation with RBCs,chronic inflammation | Radiography:Severe horizontal bone resorption | Oral hygiene instruction,Scaling/root planing,Antibiotic paste (tetracycline),Surgical excision (semiconductor laser) | Reduced gingival enlargement |
| 78 | Rapid whole-body depigmentation (skin/hair),White eyelashes/eyebrows,Reactive capillary hemangioma (face) | Wood’s light: Bright bluish-white depigmentation,Reflectance confocal microscopy,RCM:Absence of pigment cells/rings | CT: Reduced pleural effusion/tumor size | Euthyrox (75 μg/day) for hypothyroidism,No intervention for vitiligo/hemangioma | Symptoms improved |
| 79 | Multiple dome-shaped red papules (scalp, face, chest, abdomen, extremities),Ulcerated bleeding nodule (scalp) | Histopathology: Proliferating vascular,channels,fibromyxoid stroma,IHC: CD31+/CD34+/vWF+,Ki67,EBV− | NA | NA | NA |
| 80 | Multiple bright red papules (scalp, face, neck, chest, back) | Histopathology: Dilated dermal vascular channels with RBCs | NA | NA | NA |

**S 2 Detailed laboratory values,complete imaging findings, and specific drug dosing regimens**
