# Supplementary material for: Safety profile of camrelizumab: An analysis based on literature and database review
Source: PLoS One. 2026 Jul 23;21(7):e0354252. doi: 10.1371/journal.pone.0354252 (PMC13395421; doi:10.1371/journal.pone.0354252)
Supplement: Attachment — Submitted filename: Response to Reviewers.doc [file pone.0354252.s004.doc]

**Response Letter to PLOS ONE**

Manuscript Number: PONE-D-25-43837

Paper Title: Safety Profile of Camrelizumab: An Analysis Based on Literature and Database Review

We sincerely thank you for taking the time and effort to review our manuscript and for providing highly constructive and valuable suggestions. These opinions are crucial for enhancing the quality and rigor of our thesis. We have comprehensively revised the manuscript based on the suggestions of all the reviewers.We submitted 3 files, including a clean version, a version of the manuscript with key changes, and this file. In the following, we respond to each of your concerns and recommendations.

Referee 1/problem 1:**1. The novelty should be highlighted more clearly in the Introduction part, what does this add beyond existing systematic reviews of immune checkpoint inhibitors?**

Reply 1:We thank the reviewer for this important suggestion. We have revised the Introduction to more explicitly state the novelty and gap our study aims to fill. Specifically, we highlight that while systematic reviews on immune checkpoint inhibitors (ICIs) as a class exist, real-world evidence focused specifically on the safety profile of camrelizumab,is a PD-1 inhibitor with a unique adverse reaction like reactive cutaneous capillary endothelial proliferation (RCCEP)—remains scarce. Our study adds value by combining a global pharmacovigilance database analysis (WHO-VigiAccess) with a detailed systematic review of published case reports to provide a comprehensive, dual-perspective analysis of camrelizumab's ADRs.

**Change in manuscript:**The final paragraph of the Introduction has been strengthened to clearly articulate this novelty (Page 2).

Referee 1/problem 2: **99% of Vigi Access reports are from Asia, the results may not be generalizable globally. The manuscript should acknowledge under reporting bias in other regions and discuss how this might influence the safety profile.**

Reply 2: We agree with the reviewer that the geographical concentration of reports is a significant limitation affecting the generalizability of the VigiAccess results. As requested, we have now explicitly acknowledged this as a "Limitation" in the Discussion section. We discuss the potential for under-reporting in non-Asian populations and how this might lead to an incomplete understanding of the ADR profile in different ethnicities. We suggest that the observed profile may be particularly reflective of Asian patients and that future studies in more diverse populations are needed.

**Change in manuscript:**A new paragraph titled "Limitations" has been added to the Discussion section (Page 46).

Referee 1/problem 3: **The descriptive analysis of 80 case reports is rich, but the methodology for inclusion/exclusion lacks detail (e.g., was quality of reports assessed?). A PRISMA-style flow diagram is provided, but clearer justification for focusing on Chinese cases is needed.**

Reply 3:We appreciate this suggestion to enhance methodological rigor.

**Quality Assessment:**We have added a sentence to the Methods section stating that the quality of the included case reports was assessed using the standardized CARE guidelines checklist for case reports. This ensures that the included studies reported essential clinical information adequately.

**Justification for Chinese Cases:**We have provided a clearer justification in the Methods section ("Descriptive Study" subsection). We explain that camrelizumab is primarily approved and marketed in China, which naturally leads to a predominance of clinical experience and case publications from this country. Therefore, focusing on these cases provides the most relevant and concentrated real-world data currently available for this drug.

**Change in manuscript:**The Methods section now includes details on quality assessment and a better justification for the geographic focus (Page 4).

Referee 1/problem 4: **The results rely mainly on descriptive percentages. Consider additional signal detection methods (e.g., disproportionality analysis: ROR, PRR) to strengthen conclusions about specific ADRs.**

Reply 4:This is an excellent suggestion for deepening the pharmacovigilance analysis. However, due to the limitations in obtaining original data on adverse drug reactions from the WHO, we were unable to strengthen the signal of a potential causal relationship through disproportionality analysis using the ROR,PRR.

Referee 1/problem 5: **Present study lack of clinical relavence While the manuscript lists ADR frequencies, it does not sufficiently emphasize management strategies and their outcomes (e.g., how effective were steroids or immunoglobulin in preventing mortality?). A more explicit clinical recommendations section would add value.**

Reply 5:We thank the reviewer for highlighting this crucial aspect. To significantly enhance the clinical relevance, we have restructured the "Management Strategies" subsection in the Discussion. It now more explicitly links specific ADRs to their management outcomes based on our literature review. For example, we elaborate on the effectiveness of corticosteroids and IVIG in improving outcomes for myocarditis, and the multidisciplinary approach for TEN. Furthermore, we have created a new standalone section in the Conclusion titled "Clinical Recommendations," which provides concise, actionable guidance for clinicians on monitoring, patient education, and intervention timing (e.g., specific time windows for monitoring hematologic and cardiac parameters).

**Change in manuscript:**The "Management Strategies" part of the Discussion has been expanded. A new "**Clinical Recommendations**" section has been added to the Conclusion (Page 44-45).

Referee 1/problem 6: **Some terms appear inconsistent (e.g., “yyocarditis” instead of “myocarditis,” “carrelizumab” instead of “camrelizumab” in search strategy). Proofreading is needed for typographical and grammatical errors.**

Reply 6:We sincerely apologize for these typographical errors. We have conducted a thorough proofreading of the entire manuscript.

"yyocarditis" has been corrected to "myocarditis".

"carrelizumab" in the search strategy has been corrected to "camrelizumab".

All similar inconsistencies and grammatical errors have been carefully identified and corrected.

**Change in manuscript:** The entire manuscript has been proofread and corrected.

Referee 1/problem 7: **The conclusion currently repeats results. It should be sharpened to emphasize key practice implications. e.g., the importance of monitoring hematologic and cardiac toxicities at specific time windows (early vs. late onset).**

Reply 7:We agree completely. The Conclusion has been entirely rewritten to avoid mere repetition of results. It now succinctly summarizes the key findings and immediately pivots to the key clinical practice implications, as suggested by the reviewer. We explicitly emphasize the need for:

Vigilant monitoring for early-onset hematological toxicities and late-onset serious events like myocarditis and TEN.

Proactive management strategies, including the critical time windows for specific interventions.

**Change in manuscript:**The Conclusion section has been significantly revised and sharpened (Page 46).

Referee 1/problem 8: **At the outset, the manuscript is categorized as a Research Article, but the title and study design clearly indicate it is a Review. It would be more appropriate to revise the article type to Review for consistency with the content.**

Reply 8:Thank you for this correct observation. We agree that the study design, which combines a systematic literature review with a database analysis, is best classified as a Review. We have changed the manuscript category to "Review" as recommended.

Once again, we are deeply grateful for your thorough and constructive comments, which have significantly strengthened our manuscript. We hope that our revisions and responses are now satisfactory and that the manuscript is acceptable for publication.

Sincerely,

Yi Huang
