# Supplementary material for: Safety profile of camrelizumab: An analysis based on literature and database review
Source: PLoS One. 2026 Jul 23;21(7):e0354252. doi: 10.1371/journal.pone.0354252 (PMC13395421; doi:10.1371/journal.pone.0354252)
Supplement: Attachment — Submitted filename: Response to Reviewers.docx [file pone.0354252.s005.docx]

Dear Editor and Reviewers,

Thank you very much for your thorough review and valuable comments on our manuscript. We have carefully revised the manuscript according to all comments. Below is our point-by-point response:

# MAJOR COMMENTS

## 1. Novelty and Positioning of the Study

**Comment:** The Introduction should clearly state what new knowledge this study provides compared with prior ICI safety reviews. The rationale for combining VigiAccess data with a case-based literature review should be explicitly justified.

**Response:** We have completely rewritten the final paragraph of the Introduction to explicitly articulate the three innovative aspects of this study:

**Specific Revision Location:** Final paragraph of Introduction (original "Against this backdrop..." paragraph)

**Revised Text:**

"Against this backdrop, this study addresses a critical gap in current literature. While existing systematic reviews aggregate data across ICIs as a class, our work provides the first comprehensive, dual-perspective analysis of camrelizumab-specific ADRs by integrating two complementary approaches: (1) a global pharmacovigilance analysis using the WHO VigiAccess database to capture large-scale real-world ADR signals that reflect post-marketing surveillance patterns; (2) a systematic evaluation of published case reports/case series to detail clinical manifestations, temporal patterns, and management strategies. This dual-source design is deliberately chosen because VigiAccess provides quantitative signal detection across large populations but lacks clinical granularity, while case reports offer rich clinical phenotyping but are subject to publication bias. By synthesizing these complementary data streams, we achieve a more complete safety profile than either source alone. Notably, camrelizumab's unique association with reactive cutaneous capillary endothelial proliferation (RCCEP),rarely reported with other PD-1 inhibitors,underscores the necessity of drug-specific analyses beyond generic ICI reviews. Ultimately, our findings aim to optimize clinical decision-making for camrelizumab and contribute nuanced insights into the safety of PD-1 inhibitors with distinct ADR profiles."

## 2. Limitations of VigiAccess Data and Reporting Bias

**Comment:** Approximately 99% of ADR reports originate from Asia, raising concerns about geographical reporting bias. Under-reporting in non-Asian regions should be acknowledged. Implications of ethnicity, regional prescribing patterns, and regulatory approval status should be discussed.

**Response:** We have added a dedicated "Limitations and Generalizability Considerations" subsection in the Discussion:

**New Text:**

“Limitations and Generalizability Considerations
A notable limitation of this study lies in the geographical concentration of adverse drug reaction (ADR) reports within the WHO VigiAccess database. As identified, approximately 99% of camrelizumab-related reports in VigiAccess originated from Asian countries, with a predominant contribution from China, where camrelizumab was first approved and has been widely adopted in clinical practice since 2019. This regional skew raises concerns regarding the global generalizability of our pharmacovigilance findings. The observed ADR profile may therefore primarily reflect the safety experience of Asian patient populations, particularly those in East Asia, and may not fully represent the safety profile in other ethnic groups or healthcare contexts.
Several factors likely contribute to this geographical concentration: (1) camrelizumab received its first regulatory approval in China in May 2019 and was primarily marketed in Asia during the study period; (2) reporting practices and pharmacovigilance infrastructure vary significantly across regions, potentially leading to under-reporting from non-Asian countries; (3) genetic polymorphisms in drug metabolism and immune response genes may influence ADR susceptibility, though our data cannot directly test this hypothesis.
Furthermore, the VigiAccess database lacks denominator data (total number of patients exposed to camrelizumab), precluding calculation of true incidence rates and limiting our analysis to descriptive frequencies. As a passive reporting system, VigiAccess is also subject to inherent limitations including reporting bias, incomplete data fields, and inability to establish causal relationships between camrelizumab and reported events. The observed associations should be interpreted as pharmacovigilance signals requiring further validation rather than confirmed adverse reactions.
While this limitation tempers broad extrapolation, it is important to contextualize the findings within Asia's role as a key region for camrelizumab use. The data still provide valuable insights into the safety profile of the drug in its most extensively studied population. However, future research should prioritize multi-center, cross-ethnic studies integrating additional global databases (e.g., FAERS, EudraVigilance) and prospective observational cohorts to validate these observations and clarify potential racial/ethnic variations in camrelizumab-associated ADRs. Such efforts will be critical to refining risk stratification and personalized safety monitoring strategies worldwide."

## 3. Methodology of Literature-Based Case Analysis

**Comment:** Methodological transparency is insufficient. Inclusion/exclusion criteria require clearer justification. Whether the quality of included case reports was formally assessed is unclear. The rationale for focusing exclusively on Chinese cases needs clear explanation.

**Response:** We have supplemented the "Descriptive Study" subsection in the Methods section as follows:

**Specific Revisions:**

1. Explicit quality assessment tool:

The quality of the included case reports was assessed using the standardized CARE (Case report) guidelines checklist for case reports, which includes 13 core items covering title, keywords, abstract, introduction, patient information, clinical findings, timeline, diagnostic assessment, therapeutic interventions, follow-up and outcomes, discussion, patient perspective, and informed consent. Only case reports meeting at least 10 of 13 CARE criteria were included in the final analysis.

2. Explanation for geographic focus:

Camrelizumab is primarily approved and marketed in China, which consequently results in a predominance of clinical experience and case publications originating from this country. As of 2025, China accounts for >90% of global camrelizumab prescriptions. Therefore, focusing on these cases provides the most relevant and concentrated real-world data currently available for this drug, though we acknowledge this limits generalizability to other populations.

3. PRISMA flow diagram reference:

The inclusion process for case and case series studies is shown in Figure 1 (PRISMA flow diagram), which details the identification, screening, eligibility assessment, and inclusion phases.

## 4. Absence of Signal Detection or Comparative Analysis

**Comment:** The pharmacovigilance analysis is limited to descriptive statistics. No disproportionality analyses (e.g., ROR, PRR) are presented. This limitation should be explicitly stated.

**Response:** We have added the following statement at the beginning of the Results section (in addition to the Discussion):

**New Text:**

“Data Limitations Statement: It is important to note that the WHO VigiAccess database provides de-identified individual case safety reports without denominator data (total exposed population) or comparison drug data. Consequently, disproportionality analyses such as Reporting Odds Ratio (ROR), Proportional Reporting Ratio (PRR), or Information Component (IC) could not be performed. The frequencies reported here represent crude counts and proportions among reported cases, not true incidence rates or statistical signals of disproportionate reporting. All associations should be interpreted as descriptive patterns requiring further validation through controlled studies."

## 5. Clinical Relevance and Management Outcomes

**Comment:** ADR frequencies are well described, but clinical implications are insufficiently emphasized. Management strategies are mentioned but not systematically linked to outcomes. Mortality and recovery data should be interpreted more meaningfully.

**Response:** We have added a new "Clinical Recommendations" subsection in the Discussion and expanded the Management Strategies section:

**New "Clinical Recommendations" Subsection:**

Clinical Recommendations
 Based on our comprehensive analysis of camrelizumab-associated ADRs, we propose the following evidence-based recommendations to optimize clinical management:

(1) Proactive, time-window-guided monitoring:
Baseline assessment:Cardiac enzymes (troponin, CK-MB), liver function tests, thyroid function, and complete blood count should be obtained before initiation of camrelizumab.
Intensive monitoring: Heightened vigilance for ADRs is recommended during the first 8 weeks of treatment, when the majority of events occur.
Long-term surveillance: For patients on extended therapy, periodic monitoring of cardiac and endocrine function is advised given the potential for delayed-onset irAEs.

(2) Patient education and reporting:
Patients should be educated on common ADRs and instructed to report any new or worsening symptoms promptly.
Healthcare providers should maintain high index of suspicion for irAEs, particularly in patients with pre-existing autoimmune conditions.

## 6. Article Type Misclassification

**Comment:** Despite being categorized as a Research Article, the study design primarily reflects a review combined with database analysis.

**Response:** We have revised the article type to Review to better align with the content and methodology.

# MINOR COMMENTS

1. Multiple typographical and terminology errors have been corrected (e.g., "yyocarditis" → "myocarditis").
2. Drug names have been standardized throughout the manuscript.
3. Tables have been streamlined for clarity.
4. The Conclusion section has been rewritten to emphasize implications rather than repeating results.

We believe these revisions have significantly improved the manuscript. We hope the revised version is now suitable for publication. Thank you again for your valuable feedback and guidance.

Sincerely,

Yi Huang
