# Supplementary material for: Safety profile of camrelizumab: An analysis based on literature and database review
Source: PLoS One. 2026 Jul 23;21(7):e0354252. doi: 10.1371/journal.pone.0354252 (PMC13395421; doi:10.1371/journal.pone.0354252)
Supplement: Peer review history

# Peer review: Decision Letter 0

**DOI:** 10.1371/journal.pone.0354252.r001

**Authors:** Mohammed Misbah Ul Haq

19 Nov 2025

PONE-D-25-43837

Safety Profile of Camrelizumab: An Analysis Based on Literature and Database Review

PLOS ONE

Dear Dr. Huang,

Thank you for submitting your manuscript to PLOS ONE. After careful consideration, we feel that it has merit but does not fully meet PLOS ONE’s publication criteria as it currently stands. Therefore, we invite you to submit a revised version of the manuscript that addresses the points raised during the review process.

Please submit your revised manuscript by Jan 03 2026 11:59PM. If you will need more time than this to complete your revisions, please reply to this message or contact the journal office at plosone@plos.org. When you're ready to submit your revision, log on to https://www.editorialmanager.com/pone/ and select the 'Submissions Needing Revision' folder to locate your manuscript file.

Please include the following items when submitting your revised manuscript:

- A rebuttal letter that responds to each point raised by the academic editor and reviewer(s). You should upload this letter as a separate file labeled 'Response to Reviewers'.
- A marked-up copy of your manuscript that highlights changes made to the original version. You should upload this as a separate file labeled 'Revised Manuscript with Track Changes'.
- An unmarked version of your revised paper without tracked changes. You should upload this as a separate file labeled 'Manuscript'.

If you would like to make changes to your financial disclosure, please include your updated statement in your cover letter. Guidelines for resubmitting your figure files are available below the reviewer comments at the end of this letter.

If applicable, we recommend that you deposit your laboratory protocols in protocols.io to enhance the reproducibility of your results. Protocols.io assigns your protocol its own identifier (DOI) so that it can be cited independently in the future. For instructions see: https://journals.plos.org/plosone/s/submission-guidelines#loc-laboratory-protocols. Additionally, PLOS ONE offers an option for publishing peer-reviewed Lab Protocol articles, which describe protocols hosted on protocols.io. Read more information on sharing protocols at https://plos.org/protocols?utm_medium=editorial-email&utm_source=authorletters&utm_campaign=protocols.

We look forward to receiving your revised manuscript.

Kind regards,

Dr. Mohammed Misbah Ul Haq, Pharm-D

Academic Editor

PLOS ONE

Journal requirements:

When submitting your revision, we need you to address these additional requirements.

1. Please ensure that your manuscript meets PLOS ONE's style requirements, including those for file naming. The PLOS ONE style templates can be found at

https://journals.plos.org/plosone/s/file?id=wjVg/PLOSOne_formatting_sample_main_body.pdf and

https://journals.plos.org/plosone/s/file?id=ba62/PLOSOne_formatting_sample_title_authors_affiliations.pdf

2. We note that the grant information you provided in the ‘Funding Information’ and ‘Financial Disclosure’ sections do not match.

When you resubmit, please ensure that you provide the correct grant numbers for the awards you received for your study in the ‘Funding Information’ section.

3. Thank you for stating the following in the Funding Section of your manuscript:

“This study was supported by Health Research Project of Hunan Provincial Health Commission (grant number:D202313018845).”

We note that you have provided funding information that is not currently declared in your Funding Statement. However, funding information should not appear in the Acknowledgments section or other areas of your manuscript. We will only publish funding information present in the Funding Statement section of the online submission form.

Please remove any funding-related text from the manuscript and let us know how you would like to update your Funding Statement. Currently, your Funding Statement reads as follows:

“The author(s) received no specific funding for this work.”

Please include your amended statements within your cover letter; we will change the online submission form on your behalf.

4. PLOS requires an ORCID iD for the corresponding author in Editorial Manager on papers submitted after December 6th, 2016. Please ensure that you have an ORCID iD and that it is validated in Editorial Manager. To do this, go to ‘Update my Information’ (in the upper left-hand corner of the main menu), and click on the Fetch/Validate link next to the ORCID field. This will take you to the ORCID site and allow you to create a new iD or authenticate a pre-existing iD in Editorial Manager.

If the reviewer comments include a recommendation to cite specific previously published works, please review and evaluate these publications to determine whether they are relevant and should be cited. There is no requirement to cite these works unless the editor has indicated otherwise.

[Note: HTML markup is below. Please do not edit.]

Reviewers' comments:

Reviewer's Responses to Questions

**Comments to the Author**

1. Is the manuscript technically sound, and do the data support the conclusions?

The manuscript must describe a technically sound piece of scientific research with data that supports the conclusions. Experiments must have been conducted rigorously, with appropriate controls, replication, and sample sizes. The conclusions must be drawn appropriately based on the data presented.

Reviewer #1: Yes

**********

2. Has the statistical analysis been performed appropriately and rigorously?

Reviewer #1: Yes

**********

3. Have the authors made all data underlying the findings in their manuscript fully available?

The PLOS Data policy requires authors to make all data underlying the findings described in their manuscript fully available without restriction, with rare exception (please refer to the Data Availability Statement in the manuscript PDF file). The data should be provided as part of the manuscript or its supporting information, or deposited to a public repository. For example, in addition to summary statistics, the data points behind means, medians and variance measures should be available. If there are restrictions on publicly sharing data—e.g. participant privacy or use of data from a third party—those must be specified.

Reviewer #1: Yes

**********

4. Is the manuscript presented in an intelligible fashion and written in standard English?

PLOS ONE does not copyedit accepted manuscripts, so the language in submitted articles must be clear, correct, and unambiguous. Any typographical or grammatical errors should be corrected at revision, so please note any specific errors here.

Reviewer #1: Yes

**********

5. Review Comments to the Author

Please use the space provided to explain your answers to the questions above. You may also include additional comments for the author, including concerns about dual publication, research ethics, or publication ethics. (Please upload your review as an attachment if it exceeds 20,000 characters)

Reviewer #1: Dear authors.,

The manuscript entitled ‘Safety Profile of Camrelizumab: An Analysis Based on Literature and Database Review’ presents valuable work. However, to enhance readability, clinical relevance, and overall impact for the readers, the following comments should be addressed.

1. The novelty should be highlighted more clearly in the Introduction part, what does this add beyond existing systematic reviews of immune checkpoint inhibitors?

2. 99% of Vigi Access reports are from Asia, the results may not be generalizable globally. The manuscript should acknowledge under reporting bias in other regions and discuss how this might influence the safety profile.

3. The descriptive analysis of 80 case reports is rich, but the methodology for inclusion/exclusion lacks detail (e.g., was quality of reports assessed?). A PRISMA-style flow diagram is provided, but clearer justification for focusing on Chinese cases is needed.

4. The results rely mainly on descriptive percentages. Consider additional signal detection methods (e.g., disproportionality analysis: ROR, PRR) to strengthen conclusions about specific ADRs.

5. Present study lack of clinical relavence While the manuscript lists ADR frequencies, it does not sufficiently emphasize management strategies and their outcomes (e.g., how effective were steroids or immunoglobulin in preventing mortality?). A more explicit clinical recommendations section would add value

6. Some terms appear inconsistent (e.g., “yyocarditis” instead of “myocarditis,” “carrelizumab” instead of “camrelizumab” in search strategy). Proofreading is needed for typographical and grammatical errors.

7. The conclusion currently repeats results. It should be sharpened to emphasize key practice implications. e.g., the importance of monitoring hematologic and cardiac toxicities at specific time windows (early vs. late onset).

8. At the outset, the manuscript is categorized as a Research Article, but the title and study design clearly indicate it is a Review. It would be more appropriate to revise the article type to Review for consistency with the content.

**********

6. PLOS authors have the option to publish the peer review history of their article (what does this mean?). If published, this will include your full peer review and any attached files.

If you choose “no”, your identity will remain anonymous but your review may still be made public.

**Do you want your identity to be public for this peer review?**  For information about this choice, including consent withdrawal, please see our Privacy Policy.

Reviewer #1: **Yes:**  Ranakishor Pelluri Ph.D

Department of Pharmacy Practice

KL College of Pharmacy

Koneru Lakshmaiah Education Foundation

(Deemed to be University)

Vaddeswaram, Guntur-522302, India.

**********

[NOTE: If reviewer comments were submitted as an attachment file, they will be attached to this email and accessible via the submission site. Please log into your account, locate the manuscript record, and check for the action link "View Attachments". If this link does not appear, there are no attachment files.]

To ensure your figures meet our technical requirements, please review our figure guidelines: https://journals.plos.org/plosone/s/figures

You may also use PLOS’s free figure tool, NAAS, to help you prepare publication quality figures: https://journals.plos.org/plosone/s/figures#loc-tools-for-figure-preparation.

NAAS will assess whether your figures meet our technical requirements by comparing each figure against our figure specifications.

# Author comment: Author response to Decision Letter 1

**DOI:** 10.1371/journal.pone.0354252.r002

25 Dec 2025

Manuscript Number: PONE-D-25-43837

Paper Title: Safety Profile of Camrelizumab: An Analysis Based on Literature and Database Review

We sincerely thank you for taking the time and effort to review our manuscript and for providing highly constructive and valuable suggestions. These opinions are crucial for enhancing the quality and rigor of our thesis. We have comprehensively revised the manuscript based on the suggestions of all the reviewers.We submitted 3 files, including a clean version, a version of the manuscript with key changes, and this file. In the following, we respond to each of your concerns and recommendations.

Referee 1/problem 1:1. The novelty should be highlighted more clearly in the Introduction part, what does this add beyond existing systematic reviews of immune checkpoint inhibitors?

Reply 1:We thank the reviewer for this important suggestion. We have revised the Introduction to more explicitly state the novelty and gap our study aims to fill. Specifically, we highlight that while systematic reviews on immune checkpoint inhibitors (ICIs) as a class exist, real-world evidence focused specifically on the safety profile of camrelizumab,is a PD-1 inhibitor with a unique adverse reaction like reactive cutaneous capillary endothelial proliferation (RCCEP)—remains scarce. Our study adds value by combining a global pharmacovigilance database analysis (WHO-VigiAccess) with a detailed systematic review of published case reports to provide a comprehensive, dual-perspective analysis of camrelizumab's ADRs.

Change in manuscript:The final paragraph of the Introduction has been strengthened to clearly articulate this novelty (Page 2).

Referee 1/problem 2: 99% of Vigi Access reports are from Asia, the results may not be generalizable globally. The manuscript should acknowledge under reporting bias in other regions and discuss how this might influence the safety profile.

Reply 2: We agree with the reviewer that the geographical concentration of reports is a significant limitation affecting the generalizability of the VigiAccess results. As requested, we have now explicitly acknowledged this as a "Limitation" in the Discussion section. We discuss the potential for under-reporting in non-Asian populations and how this might lead to an incomplete understanding of the ADR profile in different ethnicities. We suggest that the observed profile may be particularly reflective of Asian patients and that future studies in more diverse populations are needed.

Change in manuscript:A new paragraph titled "Limitations" has been added to the Discussion section (Page 46).

Referee 1/problem 3: The descriptive analysis of 80 case reports is rich, but the methodology for inclusion/exclusion lacks detail (e.g., was quality of reports assessed?). A PRISMA-style flow diagram is provided, but clearer justification for focusing on Chinese cases is needed.

Reply 3:We appreciate this suggestion to enhance methodological rigor.

Quality Assessment:We have added a sentence to the Methods section stating that the quality of the included case reports was assessed using the standardized CARE guidelines checklist for case reports. This ensures that the included studies reported essential clinical information adequately.

Justification for Chinese Cases:We have provided a clearer justification in the Methods section ("Descriptive Study" subsection). We explain that camrelizumab is primarily approved and marketed in China, which naturally leads to a predominance of clinical experience and case publications from this country. Therefore, focusing on these cases provides the most relevant and concentrated real-world data currently available for this drug.

Change in manuscript:The Methods section now includes details on quality assessment and a better justification for the geographic focus (Page 4).

Referee 1/problem 4: The results rely mainly on descriptive percentages. Consider additional signal detection methods (e.g., disproportionality analysis: ROR, PRR) to strengthen conclusions about specific ADRs.

Reply 4:This is an excellent suggestion for deepening the pharmacovigilance analysis. However, due to the limitations in obtaining original data on adverse drug reactions from the WHO, we were unable to strengthen the signal of a potential causal relationship through disproportionality analysis using the ROR,PRR.

Referee 1/problem 5: Present study lack of clinical relavence While the manuscript lists ADR frequencies, it does not sufficiently emphasize management strategies and their outcomes (e.g., how effective were steroids or immunoglobulin in preventing mortality?). A more explicit clinical recommendations section would add value.

Reply 5:We thank the reviewer for highlighting this crucial aspect. To significantly enhance the clinical relevance, we have restructured the "Management Strategies" subsection in the Discussion. It now more explicitly links specific ADRs to their management outcomes based on our literature review. For example, we elaborate on the effectiveness of corticosteroids and IVIG in improving outcomes for myocarditis, and the multidisciplinary approach for TEN. Furthermore, we have created a new standalone section in the Conclusion titled "Clinical Recommendations," which provides concise, actionable guidance for clinicians on monitoring, patient education, and intervention timing (e.g., specific time windows for monitoring hematologic and cardiac parameters).

Change in manuscript:The "Management Strategies" part of the Discussion has been expanded. A new "Clinical Recommendations" section has been added to the Conclusion (Page 44-45).

Referee 1/problem 6: Some terms appear inconsistent (e.g., “yyocarditis” instead of “myocarditis,” “carrelizumab” instead of “camrelizumab” in search strategy). Proofreading is needed for typographical and grammatical errors.

Reply 6:We sincerely apologize for these typographical errors. We have conducted a thorough proofreading of the entire manuscript.

"yyocarditis" has been corrected to "myocarditis".

"carrelizumab" in the search strategy has been corrected to "camrelizumab".

All similar inconsistencies and grammatical errors have been carefully identified and corrected.

Change in manuscript: The entire manuscript has been proofread and corrected.

Referee 1/problem 7: The conclusion currently repeats results. It should be sharpened to emphasize key practice implications. e.g., the importance of monitoring hematologic and cardiac toxicities at specific time windows (early vs. late onset).

Reply 7:We agree completely. The Conclusion has been entirely rewritten to avoid mere repetition of results. It now succinctly summarizes the key findings and immediately pivots to the key clinical practice implications, as suggested by the reviewer. We explicitly emphasize the need for:

Vigilant monitoring for early-onset hematological toxicities and late-onset serious events like myocarditis and TEN.

Proactive management strategies, including the critical time windows for specific interventions.

Change in manuscript:The Conclusion section has been significantly revised and sharpened (Page 46).

Referee 1/problem 8: At the outset, the manuscript is categorized as a Research Article, but the title and study design clearly indicate it is a Review. It would be more appropriate to revise the article type to Review for consistency with the content.

Reply 8:Thank you for this correct observation. We agree that the study design, which combines a systematic literature review with a database analysis, is best classified as a Review. We have changed the manuscript category to "Review" as recommended.

Once again, we are deeply grateful for your thorough and constructive comments, which have significantly strengthened our manuscript. We hope that our revisions and responses are now satisfactory and that the manuscript is acceptable for publication.

Sincerely,

Yi Huang

Attachment Submitted filename: Response to Reviewers.doc

# Peer review: Decision Letter 1

**DOI:** 10.1371/journal.pone.0354252.r003

**Authors:** Mohammed Misbah Ul Haq

15 Feb 2026

PONE-D-25-43837R1

Safety Profile of Camrelizumab: An Analysis Based on Literature and Database Review

PLOS One

Dear Dr. Huang,

Thank you for submitting your manuscript to PLOS ONE. After careful consideration, we feel that it has merit but does not fully meet PLOS ONE’s publication criteria as it currently stands. Therefore, we invite you to submit a revised version of the manuscript that addresses the points raised during the review process.

Please submit your revised manuscript by Apr 01 2026 11:59PM. If you will need more time than this to complete your revisions, please reply to this message or contact the journal office at plosone@plos.org. When you're ready to submit your revision, log on to https://www.editorialmanager.com/pone/ and select the 'Submissions Needing Revision' folder to locate your manuscript file.

Please include the following items when submitting your revised manuscript:

- A letter that responds to each point raised by the academic editor and reviewer(s). You should upload this letter as a separate file labeled 'Response to Reviewers'.
- A marked-up copy of your manuscript that highlights changes made to the original version. You should upload this as a separate file labeled 'Revised Manuscript with Track Changes'.
- An unmarked version of your revised paper without tracked changes. You should upload this as a separate file labeled 'Manuscript'.

If you would like to make changes to your financial disclosure, please include your updated statement in your cover letter. Guidelines for resubmitting your figure files are available below the reviewer comments at the end of this letter.

If applicable, we recommend that you deposit your laboratory protocols in protocols.io to enhance the reproducibility of your results. Protocols.io assigns your protocol its own identifier (DOI) so that it can be cited independently in the future. For instructions see: https://journals.plos.org/plosone/s/submission-guidelines#loc-laboratory-protocols. Additionally, PLOS ONE offers an option for publishing peer-reviewed Lab Protocol articles, which describe protocols hosted on protocols.io. Read more information on sharing protocols at https://plos.org/protocols?utm_medium=editorial-email&utm_source=authorletters&utm_campaign=protocols.

We look forward to receiving your revised manuscript.

Kind regards,

Mohammed Misbah Ul Haq, Pharm-D

Academic Editor

PLOS One

Journal Requirements:

If the reviewer comments include a recommendation to cite specific previously published works, please review and evaluate these publications to determine whether they are relevant and should be cited. There is no requirement to cite these works unless the editor has indicated otherwise.

Additional Editor Comments :

Reviewer Comments

General Assessment

This manuscript presents a combined analysis of real-world pharmacovigilance data from the WHO-VigiAccess database and a descriptive review of published case reports on camrelizumab-associated adverse drug reactions (ADRs). The topic is clinically relevant, particularly given the increasing use of camrelizumab and its distinct toxicity profile (e.g., RCCEP). However, several methodological, interpretative, and presentation issues must be addressed before the manuscript can be considered for publication. Substantial revisions are required to improve scientific rigor, transparency, and clinical relevance.

Major Comments

1. Novelty and Positioning of the Study

While the manuscript addresses camrelizumab-related adverse events, the novel contribution beyond existing reviews of immune checkpoint inhibitors (ICIs) is not sufficiently articulated.

• The Introduction should clearly state what new knowledge this study provides compared with prior ICI safety reviews and post-marketing surveillance analyses.

• The rationale for combining VigiAccess data with a case-based literature review should be explicitly justified.

Recommendation: Strengthen the final paragraph of the Introduction to clearly define the knowledge gap and the specific added value of this dual-source approach.

2. Limitations of VigiAccess Data and Reporting Bias

Approximately 99% of ADR reports originate from Asia, which raises concerns about geographical reporting bias and limited global generalizability.

• Under-reporting in non-Asian regions should be explicitly acknowledged.

• The implications of ethnicity, regional prescribing patterns, and regulatory approval status on the observed safety profile should be discussed.

Recommendation: Add a dedicated Limitations subsection in the Discussion addressing reporting bias, lack of denominator data, and inability to infer causality.

3. Methodology of Literature-Based Case Analysis

The descriptive analysis of 80 published cases is detailed, but methodological transparency is insufficient.

• Inclusion and exclusion criteria require clearer justification.

• It is unclear whether the quality of included case reports was formally assessed.

• The rationale for focusing exclusively on Chinese cases, although plausible, needs to be clearly explained in the Methods.

Recommendation:

• Specify whether a standardized tool (e.g., CARE guidelines) was used to assess case report quality.

• Justify the geographic focus based on drug approval status and availability.

• Ensure the PRISMA flow diagram is clearly referenced and explained in the text.

4. Absence of Signal Detection or Comparative Analysis

The pharmacovigilance analysis is limited to descriptive statistics.

• No disproportionality analyses (e.g., ROR, PRR) are presented to support potential safety signals.

• While data access limitations may exist, this should be explicitly stated.

Recommendation:

If disproportionality analysis is not feasible, clearly acknowledge this limitation and avoid language suggesting causal associations.

5. Clinical Relevance and Management Outcomes

Although ADR frequencies are well described, clinical implications are insufficiently emphasized.

• Management strategies (e.g., corticosteroids, IVIG) are mentioned but not systematically linked to outcomes.

• Mortality and recovery data should be interpreted in a more clinically meaningful manner.

Recommendation:

Expand the Discussion to explicitly connect specific ADRs with management approaches and outcomes, and consider adding a short Clinical Recommendations subsection summarizing actionable points for clinicians.

6. Article Type Misclassification

Despite being categorized as a Research Article, the study design primarily reflects a review combined with database analysis.

Recommendation:

Revise the article type to Review (or clarify with the editorial office) to better align with the content and methodology.

Minor Comments

1. Multiple typographical and terminology errors are present (e.g., “yyocarditis” instead of “myocarditis”).

2. Drug names should be used consistently throughout (e.g., camrelizumab vs. alternative spellings).

3. Some tables are overly detailed and may benefit from streamlining or relocation to supplementary material.

4. The Conclusion section currently repeats results rather than emphasizing implications.

Recommendation:

Careful proofreading is required, and the Conclusion should be rewritten to highlight key safety concerns, monitoring priorities, and future research needs.

Overall Recommendation

Major Revision

The manuscript addresses an important clinical topic but requires substantial revision to improve methodological clarity, acknowledge limitations appropriately, strengthen clinical interpretation, and ensure consistency with journal standards.

[Note: HTML markup is below. Please do not edit.]

Reviewers' comments:

Reviewer's Responses to Questions

**Comments to the Author**

1. If the authors have adequately addressed your comments raised in a previous round of review and you feel that this manuscript is now acceptable for publication, you may indicate that here to bypass the “Comments to the Author” section, enter your conflict of interest statement in the “Confidential to Editor” section, and submit your "Accept" recommendation.

Reviewer #2: (No Response)

**********

2. Is the manuscript technically sound, and do the data support the conclusions?

The manuscript must describe a technically sound piece of scientific research with data that supports the conclusions. Experiments must have been conducted rigorously, with appropriate controls, replication, and sample sizes. The conclusions must be drawn appropriately based on the data presented.

Reviewer #2: (No Response)

**********

3. Has the statistical analysis been performed appropriately and rigorously?

Reviewer #2: (No Response)

**********

4. Have the authors made all data underlying the findings in their manuscript fully available?

The PLOS Data policy requires authors to make all data underlying the findings described in their manuscript fully available without restriction, with rare exception (please refer to the Data Availability Statement in the manuscript PDF file). The data should be provided as part of the manuscript or its supporting information, or deposited to a public repository. For example, in addition to summary statistics, the data points behind means, medians and variance measures should be available. If there are restrictions on publicly sharing data—e.g. participant privacy or use of data from a third party—those must be specified.

Reviewer #2: (No Response)

**********

5. Is the manuscript presented in an intelligible fashion and written in standard English?

PLOS ONE does not copyedit accepted manuscripts, so the language in submitted articles must be clear, correct, and unambiguous. Any typographical or grammatical errors should be corrected at revision, so please note any specific errors here.

Reviewer #2: (No Response)

**********

6. Review Comments to the Author

Please use the space provided to explain your answers to the questions above. You may also include additional comments for the author, including concerns about dual publication, research ethics, or publication ethics. (Please upload your review as an attachment if it exceeds 20,000 characters)

Reviewer #2: (No Response)

**********

7. PLOS authors have the option to publish the peer review history of their article (what does this mean?). If published, this will include your full peer review and any attached files.

If you choose “no”, your identity will remain anonymous but your review may still be made public.

**Do you want your identity to be public for this peer review?**  For information about this choice, including consent withdrawal, please see our Privacy Policy.

Reviewer #2: **Yes:**  Dr. Hammad Baig

**********

[NOTE: If reviewer comments were submitted as an attachment file, they will be attached to this email and accessible via the submission site. Please log into your account, locate the manuscript record, and check for the action link "View Attachments". If this link does not appear, there are no attachment files.]

To ensure your figures meet our technical requirements, please review our figure guidelines: https://journals.plos.org/plosone/s/figures

You may also use PLOS’s free figure tool, NAAS, to help you prepare publication quality figures: https://journals.plos.org/plosone/s/figures#loc-tools-for-figure-preparation.

NAAS will assess whether your figures meet our technical requirements by comparing each figure against our figure specifications.

# Author comment: Author response to Decision Letter 2

**DOI:** 10.1371/journal.pone.0354252.r004

9 Apr 2026

Dear Editor and Reviewers,

Thank you very much for your thorough review and valuable comments on our manuscript. We have carefully revised the manuscript according to all comments. Below is our point-by-point response:

MAJOR COMMENTS

1. Novelty and Positioning of the Study

Comment: The Introduction should clearly state what new knowledge this study provides compared with prior ICI safety reviews. The rationale for combining VigiAccess data with a case-based literature review should be explicitly justified.

Response: We have completely rewritten the final paragraph of the Introduction to explicitly articulate the three innovative aspects of this study:

Specific Revision Location: Final paragraph of Introduction (original "Against this backdrop..." paragraph)

Revised Text:

"Against this backdrop, this study addresses a critical gap in current literature. While existing systematic reviews aggregate data across ICIs as a class, our work provides the first comprehensive, dual-perspective analysis of camrelizumab-specific ADRs by integrating two complementary approaches: (1) a global pharmacovigilance analysis using the WHO VigiAccess database to capture large-scale real-world ADR signals that reflect post-marketing surveillance patterns; (2) a systematic evaluation of published case reports/case series to detail clinical manifestations, temporal patterns, and management strategies. This dual-source design is deliberately chosen because VigiAccess provides quantitative signal detection across large populations but lacks clinical granularity, while case reports offer rich clinical phenotyping but are subject to publication bias. By synthesizing these complementary data streams, we achieve a more complete safety profile than either source alone. Notably, camrelizumab's unique association with reactive cutaneous capillary endothelial proliferation (RCCEP),rarely reported with other PD-1 inhibitors,underscores the necessity of drug-specific analyses beyond generic ICI reviews. Ultimately, our findings aim to optimize clinical decision-making for camrelizumab and contribute nuanced insights into the safety of PD-1 inhibitors with distinct ADR profiles."

2. Limitations of VigiAccess Data and Reporting Bias

Comment: Approximately 99% of ADR reports originate from Asia, raising concerns about geographical reporting bias. Under-reporting in non-Asian regions should be acknowledged. Implications of ethnicity, regional prescribing patterns, and regulatory approval status should be discussed.

Response: We have added a dedicated "Limitations and Generalizability Considerations" subsection in the Discussion:

New Text:

“Limitations and Generalizability Considerations

A notable limitation of this study lies in the geographical concentration of adverse drug reaction (ADR) reports within the WHO VigiAccess database. As identified, approximately 99% of camrelizumab-related reports in VigiAccess originated from Asian countries, with a predominant contribution from China, where camrelizumab was first approved and has been widely adopted in clinical practice since 2019. This regional skew raises concerns regarding the global generalizability of our pharmacovigilance findings. The observed ADR profile may therefore primarily reflect the safety experience of Asian patient populations, particularly those in East Asia, and may not fully represent the safety profile in other ethnic groups or healthcare contexts.

Several factors likely contribute to this geographical concentration: (1) camrelizumab received its first regulatory approval in China in May 2019 and was primarily marketed in Asia during the study period; (2) reporting practices and pharmacovigilance infrastructure vary significantly across regions, potentially leading to under-reporting from non-Asian countries; (3) genetic polymorphisms in drug metabolism and immune response genes may influence ADR susceptibility, though our data cannot directly test this hypothesis.

Furthermore, the VigiAccess database lacks denominator data (total number of patients exposed to camrelizumab), precluding calculation of true incidence rates and limiting our analysis to descriptive frequencies. As a passive reporting system, VigiAccess is also subject to inherent limitations including reporting bias, incomplete data fields, and inability to establish causal relationships between camrelizumab and reported events. The observed associations should be interpreted as pharmacovigilance signals requiring further validation rather than confirmed adverse reactions.

While this limitation tempers broad extrapolation, it is important to contextualize the findings within Asia's role as a key region for camrelizumab use. The data still provide valuable insights into the safety profile of the drug in its most extensively studied population. However, future research should prioritize multi-center, cross-ethnic studies integrating additional global databases (e.g., FAERS, EudraVigilance) and prospective observational cohorts to validate these observations and clarify potential racial/ethnic variations in camrelizumab-associated ADRs. Such efforts will be critical to refining risk stratification and personalized safety monitoring strategies worldwide."

3. Methodology of Literature-Based Case Analysis

Comment: Methodological transparency is insufficient. Inclusion/exclusion criteria require clearer justification. Whether the quality of included case reports was formally assessed is unclear. The rationale for focusing exclusively on Chinese cases needs clear explanation.

Response: We have supplemented the "Descriptive Study" subsection in the Methods section as follows:

Specific Revisions:

1. Explicit quality assessment tool:

The quality of the included case reports was assessed using the standardized CARE (Case report) guidelines checklist for case reports, which includes 13 core items covering title, keywords, abstract, introduction, patient information, clinical findings, timeline, diagnostic assessment, therapeutic interventions, follow-up and outcomes, discussion, patient perspective, and informed consent. Only case reports meeting at least 10 of 13 CARE criteria were included in the final analysis.

2. Explanation for geographic focus:

Camrelizumab is primarily approved and marketed in China, which consequently results in a predominance of clinical experience and case publications originating from this country. As of 2025, China accounts for >90% of global camrelizumab prescriptions. Therefore, focusing on these cases provides the most relevant and concentrated real-world data currently available for this drug, though we acknowledge this limits generalizability to other populations.

3. PRISMA flow diagram reference:

The inclusion process for case and case series studies is shown in Figure 1 (PRISMA flow diagram), which details the identification, screening, eligibility assessment, and inclusion phases.

4. Absence of Signal Detection or Comparative Analysis

Comment: The pharmacovigilance analysis is limited to descriptive statistics. No disproportionality analyses (e.g., ROR, PRR) are presented. This limitation should be explicitly stated.

Response: We have added the following statement at the beginning of the Results section (in addition to the Discussion):

New Text:

“Data Limitations Statement: It is important to note that the WHO VigiAccess database provides de-identified individual case safety reports without denominator data (total exposed population) or comparison drug data. Consequently, disproportionality analyses such as Reporting Odds Ratio (ROR), Proportional Reporting Ratio (PRR), or Information Component (IC) could not be performed. The frequencies reported here represent crude counts and proportions among reported cases, not true incidence rates or statistical signals of disproportionate reporting. All associations should be interpreted as descriptive patterns requiring further validation through controlled studies."

5. Clinical Relevance and Management Outcomes

Comment: ADR frequencies are well described, but clinical implications are insufficiently emphasized. Management strategies are mentioned but not systematically linked to outcomes. Mortality and recovery data should be interpreted more meaningfully.

Response: We have added a new "Clinical Recommendations" subsection in the Discussion and expanded the Management Strategies section:

New "Clinical Recommendations" Subsection:

Clinical Recommendations

Based on our comprehensive analysis of camrelizumab-associated ADRs, we propose the following evidence-based recommendations to optimize clinical management:

(1) Proactive, time-window-guided monitoring:

Baseline assessment:Cardiac enzymes (troponin, CK-MB), liver function tests, thyroid function, and complete blood count should be obtained before initiation of camrelizumab.

Intensive monitoring: Heightened vigilance for ADRs is recommended during the first 8 weeks of treatment, when the majority of events occur.

Long-term surveillance: For patients on extended therapy, periodic monitoring of cardiac and endocrine function is advised given the potential for delayed-onset irAEs.

(2) Patient education and reporting:

Patients should be educated on common ADRs and instructed to report any new or worsening symptoms promptly.

Healthcare providers should maintain high index of suspicion for irAEs, particularly in patients with pre-existing autoimmune conditions.

6. Article Type Misclassification

Comment: Despite being categorized as a Research Article, the study design primarily reflects a review combined with database analysis.

Response: We have revised the article type to Review to better align with the content and methodology.

MINOR COMMENTS

1. Multiple typographical and terminology errors have been corrected (e.g., "yyocarditis" → "myocarditis").

2. Drug names have been standardized throughout the manuscript.

3. Tables have been streamlined for clarity.

4. The Conclusion section has been rewritten to emphasize implications rather than repeating results.

We believe these revisions have significantly improved the manuscript. We hope the revised version is now suitable for publication. Thank you again for your valuable feedback and guidance.

Sincerely,

Yi Huang

Attachment Submitted filename: Response to Reviewers.docx

# Peer review: Decision Letter 2

**DOI:** 10.1371/journal.pone.0354252.r005

**Authors:** Mohammed Misbah Ul Haq

7 Jul 2026

Safety Profile of Camrelizumab: An Analysis Based on Literature and Database Review

PONE-D-25-43837R2

Dear Dr. Yi Huang,

We’re pleased to inform you that your manuscript has been judged scientifically suitable for publication and will be formally accepted for publication once it meets all outstanding technical requirements.

Within one week, you’ll receive an e-mail detailing the required amendments. When these have been addressed, you’ll receive a formal acceptance letter and your manuscript will be scheduled for publication.

An invoice will be generated when your article is formally accepted. Please note, if your institution has a publishing partnership with PLOS and your article meets the relevant criteria, all or part of your publication costs will be covered. Please make sure your user information is up-to-date by logging into Editorial Manager at Editorial Manager® and clicking the ‘Update My Information' link at the top of the page. For questions related to billing, please contact billing support.

If your institution or institutions have a press office, please notify them about your upcoming paper to help maximize its impact. If they’ll be preparing press materials, please inform our press team as soon as possible -- no later than 48 hours after receiving the formal acceptance. Your manuscript will remain under strict press embargo until 2 pm Eastern Time on the date of publication. For more information, please contact onepress@plos.org.

Kind regards,

Dr. Mohammed Misbah Ul Haq, Pharm-D

Academic Editor

PLOS One

Additional Editor Comments:

Both reviewers recommend acceptance and agree that the authors have satisfactorily addressed all substantive concerns raised during previous review rounds. The manuscript is technically sound, clearly presented, and the data support the conclusions. Reviewer 4 suggested a few minor editorial improvements (typographical correction and brief additions to the Discussion regarding comparison with other PD-1 inhibitors and Camrelizumab dosing), which do not affect the scientific validity of the manuscript. I recommend acceptance.

Reviewers' comments:

Reviewer's Responses to Questions

**Comments to the Author**

1. If the authors have adequately addressed your comments raised in a previous round of review and you feel that this manuscript is now acceptable for publication, you may indicate that here to bypass the “Comments to the Author” section, enter your conflict of interest statement in the “Confidential to Editor” section, and submit your "Accept" recommendation.

Reviewer #3: All comments have been addressed

Reviewer #4: All comments have been addressed

**********

2. Is the manuscript technically sound, and do the data support the conclusions?

The manuscript must describe a technically sound piece of scientific research with data that supports the conclusions. Experiments must have been conducted rigorously, with appropriate controls, replication, and sample sizes. The conclusions must be drawn appropriately based on the data presented.

Reviewer #3: Yes

Reviewer #4: Yes

**********

3. Has the statistical analysis been performed appropriately and rigorously?

Reviewer #3: Yes

Reviewer #4: Yes

**********

4. Have the authors made all data underlying the findings in their manuscript fully available?

The PLOS Data policy requires authors to make all data underlying the findings described in their manuscript fully available without restriction, with rare exception (please refer to the Data Availability Statement in the manuscript PDF file). The data should be provided as part of the manuscript or its supporting information, or deposited to a public repository. For example, in addition to summary statistics, the data points behind means, medians and variance measures should be available. If there are restrictions on publicly sharing data—e.g. participant privacy or use of data from a third party—those must be specified.

Reviewer #3: Yes

Reviewer #4: Yes

**********

5. Is the manuscript presented in an intelligible fashion and written in standard English?

PLOS ONE does not copyedit accepted manuscripts, so the language in submitted articles must be clear, correct, and unambiguous. Any typographical or grammatical errors should be corrected at revision, so please note any specific errors here.

Reviewer #3: Yes

Reviewer #4: Yes

**********

6. Review Comments to the Author

Please use the space provided to explain your answers to the questions above. You may also include additional comments for the author, including concerns about dual publication, research ethics, or publication ethics. (Please upload your review as an attachment if it exceeds 20,000 characters)

Reviewer #3: General Comments:

The authors have done a commendable job in revising the manuscript. The transition from a "Research Article" to a "Review" better reflects the study's nature, which relies on the WHO-VigiAccess database and a systematic literature review. The dual-perspective approach provides a comprehensive overview of the safety profile of Camrelizumab, which is particularly valuable for clinicians managing patients on this specific PD-1 inhibitor.

Specific Strengths:

Technical Soundness: The methodology is now clearly defined and appropriate for a pharmacovigilance study. Combining large-scale database trends with detailed clinical case reports allows for a more nuanced understanding of both common and rare adverse drug reactions (ADRs).

Clinical Utility: The "Time-to-onset" analysis and the subsequent recommendations for intensive monitoring during the first 8 weeks of treatment provide actionable insights for clinical practice.

Data Clarity: The tables have been significantly improved and streamlined, making the distribution of ADRs across different system organ classes (SOC) easy to interpret.

Language and Presentation: The manuscript is now well-written in standard academic English. The authors have successfully addressed previous typographical and terminological errors (e.g., correcting "myocarditis").

Ethics and Integrity:

The study appears to adhere to publication ethics. The use of the VigiAccess public database and published literature does not raise privacy concerns, and the authors have appropriately cited their sources. There are no concerns regarding dual publication.

Conclusion:

In its current form, the manuscript meets the standards of PLOS ONE. It provides a rigorous analysis of real-world safety data for Camrelizumab, filling a gap in the existing literature. I recommend the manuscript for publication.

Reviewer #4: The manuscript entitled, " Safety Profile of Camrelizumab: An Analysis Based on Literature and Database

Review" has been revised thoroughly by the authors incorporating the changes as per the recommendations and comments of the reviewers. Further, supplementary data is also organised as suggested. Overall, the quality of the manuscript has improved. Minor observations include: 1.In the table 1- category under sex and age-to be marked as unknown (in place of unknow). 2. Supporting literature and content regarding safety profiles of other PD-I inhibitors for comparison may be given in the discussion under management strategies. 3. Relevant information on dose of Camrelizumaband its effects is lacking.

In view of this, it is recommended that the manuscript may be considered at editorial level for acceptance after receiving author's response addressing the minor comments mentioned.

**********

7. PLOS authors have the option to publish the peer review history of their article (what does this mean?). If published, this will include your full peer review and any attached files.

If you choose “no”, your identity will remain anonymous but your review may still be made public.

**Do you want your identity to be public for this peer review?**  For information about this choice, including consent withdrawal, please see our Privacy Policy.

Reviewer #3: **Yes:**  Yahya Ali Abdulkareem Abodea

Reviewer #4: No

**********

# Editor report: Acceptance letter

**DOI:** 10.1371/journal.pone.0354252.r006

**Authors:** Mohammed Misbah Ul Haq

PONE-D-25-43837R2

PLOS One

Dear Dr. Huang,

I'm pleased to inform you that your manuscript has been deemed suitable for publication in PLOS One. Congratulations! Your manuscript is now being handed over to our production team.

At this stage, our production department will prepare your paper for publication. This includes ensuring the following:

* All references, tables, and figures are properly cited

* All relevant supporting information is included in the manuscript submission,

* There are no issues that prevent the paper from being properly typeset

You will receive further instructions from the production team, including instructions on how to review your proof when it is ready. Please keep in mind that we are working through a large volume of accepted articles, so please give us a few days to review your paper and let you know the next and final steps.

Lastly, if your institution or institutions have a press office, please let them know about your upcoming paper now to help maximize its impact. If they'll be preparing press materials, please inform our press team within the next 48 hours. Your manuscript will remain under strict press embargo until 2 pm Eastern Time on the date of publication. For more information, please contact onepress@plos.org.

You will receive an invoice from PLOS for your publication fee after your manuscript has reached the completed accept phase. If you receive an email requesting payment before acceptance or for any other service, this may be a phishing scheme. Learn how to identify phishing emails and protect your accounts at https://explore.plos.org/phishing.

If we can help with anything else, please email us at customercare@plos.org.

Thank you for submitting your work to PLOS ONE and supporting open access.

Kind regards,

PLOS ONE Editorial Office Staff

on behalf of

Dr. Mohammed Misbah Ul Haq

Academic Editor

PLOS One
